# Years of life gained by multifactorial intervention in patients with type 2 diabetes mellitus and microalbuminuria: 21 years follow-up on the Steno-2 randomised trial

**DOI:** 10.1007/s00125-016-4065-6

**Published:** 2016-08-16

**Authors:** Peter Gæde, Jens Oellgaard, Bendix Carstensen, Peter Rossing, Henrik Lund-Andersen, Hans-Henrik Parving, Oluf Pedersen

**Affiliations:** 1 Department of Cardiology and Endocrinology, Slagelse Hospital, Slagelse, Denmark; 2 Institute for Regional Health Research, University of Southern Denmark, Odense, Denmark; 3 Steno Diabetes Center, Gentofte, Denmark; 4 Faculty of Health, Aarhus University, Aarhus, Denmark; 5 Faculty of Health Sciences, University of Copenhagen, Copenhagen, Denmark; 6 Capital Region Eye Clinic, Copenhagen, Denmark; 7 Department of Medical Endocrinology, Rigshospitalet, Copenhagen, Denmark; 8 Novo Nordisk Foundation Center for Basic Metabolic Research, Section of Metabolic Genetics, Faculty of Health and Medical Sciences, University of Copenhagen, Universitetsparken 1, DK-2100 Copenhagen, Denmark

**Keywords:** Albuminuria, Cardiovascular disease, Diabetes complications, Diabetes mellitus, type 2, Diabetic nephropathy, Diabetic neuropathy, Diabetic retinopathy, Follow-up studies, Humans

## Abstract

**Aims/hypothesis:**

The aim of this work was to study the potential long-term impact of a 7.8 years intensified, multifactorial intervention in patients with type 2 diabetes mellitus and microalbuminuria in terms of gained years of life and years free from incident cardiovascular disease.

**Methods:**

The original intervention (mean treatment duration 7.8 years) involved 160 patients with type 2 diabetes and microalbuminuria who were randomly assigned (using sealed envelopes) to receive either conventional therapy or intensified, multifactorial treatment including both behavioural and pharmacological approaches. After 7.8 years the study continued as an observational follow-up with all patients receiving treatment as for the original intensive-therapy group. The primary endpoint of this follow-up 21.2 years after intervention start was difference in median survival time between the original treatment groups with and without incident cardiovascular disease. Non-fatal endpoints and causes of death were adjudicated by an external endpoint committee blinded for treatment allocation.

**Results:**

Thirty-eight intensive-therapy patients vs 55 conventional-therapy patients died during follow-up (HR 0.55 [95% CI 0.36, 0.83], *p* = 0.005). The patients in the intensive-therapy group survived for a median of 7.9 years longer than the conventional-therapy group patients. Median time before first cardiovascular event after randomisation was 8.1 years longer in the intensive-therapy group (*p* = 0.001). The hazard for all microvascular complications was decreased in the intensive-therapy group in the range 0.52 to 0.67, except for peripheral neuropathy (HR 1.12).

**Conclusions/interpretation:**

At 21.2 years of follow-up of 7.8 years of intensified, multifactorial, target-driven treatment of type 2 diabetes with microalbuminuria, we demonstrate a median of 7.9 years of gain of life. The increase in lifespan is matched by time free from incident cardiovascular disease.

**Trial registration:**

ClinicalTrials.gov registration no. NCT00320008.

**Funding:**

The study was funded by an unrestricted grant from Novo Nordisk A/S.

**Electronic supplementary material:**

The online version of this article (doi:10.1007/s00125-016-4065-6) contains peer-reviewed but unedited supplementary material, which is available to authorised users.

## Introduction

Type 2 diabetes mellitus is a complex disorder often featuring adiposity, hypertension, dyslipidaemia and increased blood platelet aggregation in addition to hyperglycaemia, giving rise to an increased risk of macro- and microvascular damage and reduced life expectancy. Over the past two decades, a number of single-risk-factor intervention and post-trial studies have provided evidence for recommending multifactorial treatment of this common disorder (as reviewed in [1]). Still, patients with type 2 diabetes mellitus have increased risk for early mortality [2] and may suffer from multiple organ damage with recurrent event rates of up to 15% per year during follow-up of various trials [3–8] even though recent surveys suggest favourable changes in diabetes-related complications including death [9, 10].

Therefore, this follow-up, a randomised trial of intensified vs standard multifactorial intervention for 7.8 years in patients with type 2 diabetes mellitus and microalbuminuria, was designed to address the median differences in lifespan with and without incident cardiovascular events between the originally intensified vs conventionally treated patients. We use the term ‘years of life gained’ to emphasise the decreased life expectancy in patients with type 2 diabetes mellitus and microalbuminuria.

In the present follow-up study the patients were observed for a median of 13.2 years after the formal interventional part of the study ended.

## Methods

### Study design

The Steno-2 study was initiated in 1993, enrolling 160 Danish patients of European descent with type 2 diabetes mellitus and microalbuminuria [11–13]. The design and methods for patient inclusion, randomisation, treatment and initial follow-up have been reported in detail previously [11–13].

Briefly, patients were randomised 1:1, stratified in blocks by sex, age, known diabetes duration and urinary albumin excretion rate (<100 mg/day vs >100 mg/day) using sealed envelopes, to receive either conventional multifactorial treatment with treatment goals at all times according to existing national guidelines or to receive intensified, multifactorial treatment targeting co-existing risk factors for late diabetic complications. Eighty patients were assigned to each group. Conventional-therapy patients were followed by their general practitioner, but at all times had the opportunity of being referred to specialist treatment. The treatment in the intensive-therapy group at a specialised diabetes clinic was target-driven with stepwise implementation of both behavioural and pharmacological treatment following a structured approach. Treatment goals for the groups are shown in Section 1 of the electronic supplementary material (ESM) in ESM Table 1.

Patients completed up to six study visits at Steno Diabetes Center, namely at baseline and after an average of 1.9, 3.8, 7.8 and 13.3 years, respectively, and at the termination visit after 21.2 years.

### Study population

**Fig. 1:**
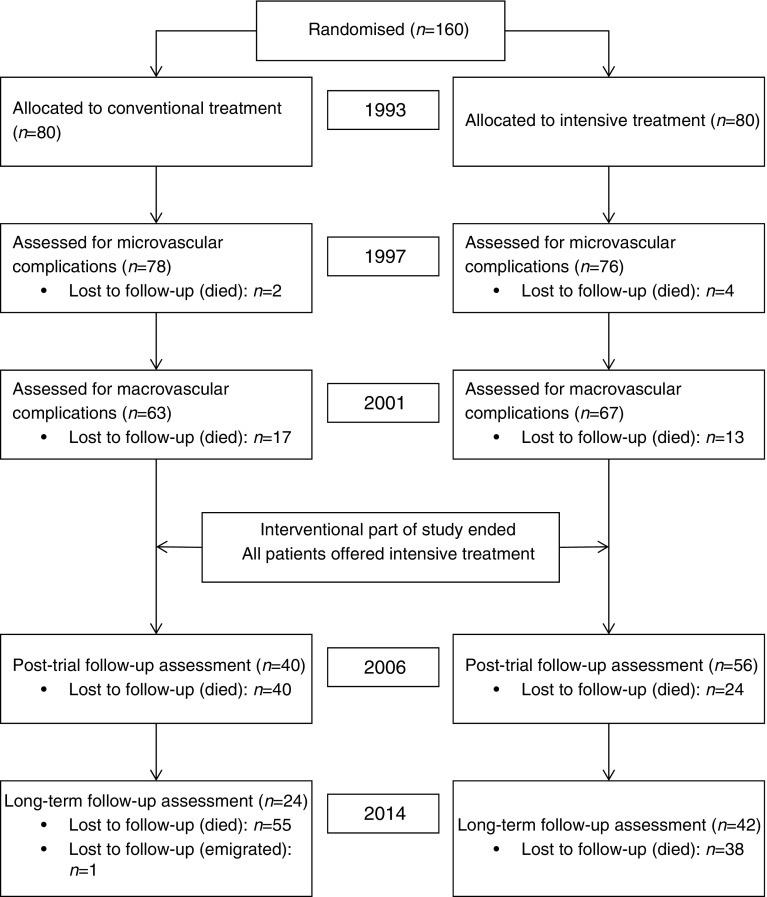
Consort diagram of patient flow throughout the entire observation period. Procedures for enrolment and randomisation are described in [11]. Numbers lost to follow-up are cumulative

Of the 160 patients originally enrolled in the Steno-2 study, 130 completed the interventional part of the study. Patient flow is depicted in Fig. 1. All patients completing the interventional part of the study provided written informed consent to continue participation in the observational post-trial follow-up. In the entire follow-up period, one patient in the conventional group and none in the intensive group were lost to follow-up. The loss was due to emigration and data collection on that patient terminated in 2007. All other patients were followed to death or study termination.

Due to ethical concerns, following the marked risk reductions found with intensive therapy after 7.8 years, all patients were subsequently offered intensified multifactorial treatment according to the original protocol using targets resembling current EASD/ESC and ADA guidelines [14, 15].

The protocol for the follow-up trial was in accordance with the declaration of Helsinki and approved by the local ethics committee (Ethics committee, Capital Region of Denmark; protocol ID number: H-KA-99035-GS, add. 41104) and by the Danish Data Protection Agency (J.Nr. 2015-41-4042).

### Procedures, measurements and endpoints

The follow-up investigations were initiated to investigate the difference in median time to 50% mortality in each of the two original treatment groups. This follow-up study was done as an amendment to the original study protocol.

The primary endpoint for the present study was the difference between the two treatment groups in the survival time after randomisation without and with cardiovascular disease (CVD). The secondary endpoint was defined as a composite of cardiovascular events (time to incident CVD and number of cardiovascular events) as well as mortality and CVD rates. The tertiary endpoints were rate of incident diabetic nephropathy, rate of end-stage renal disease and the development or progression of diabetic retinopathy and neuropathy.

For the follow-up examinations, all living patients were invited to an outpatient clinic visit assessing anthropometric, biochemical and physiological variables as well as detailed status on micro- and macrovascular complications. The examinations were conducted by a single laboratory technician who was unaware of the original randomisation assignments.

Supplementary clinical evaluations were collected from nationwide registries when not obtainable at the study visit. All measurement methods were in accordance with previous follow-up protocols [13].

For endpoint assessment, all patients were tracked by their unique, static Central Personal Registration number using the Danish Civil Registration System. All hospital contacts diagnosed to possibly fulfil the pre-specified criteria (see ESM Section 2) or relating to the criteria (including ICD-10 [www.who.int/classifications/icd/en/] observation-diagnoses [DZ0.3x] and symptom-diagnoses [e.g. DR0.74, chest pain]) were extracted from the Danish National Patient Registry and used as cases for the endpoint committee. Complete health records for cases were obtained from nationwide electronic health records and adjudicated for endpoints by an external committee masked for patients’ original treatment allocation. The registries have proven highly valid and complete [16, 17]. Hospitals report mandatorily to the registries and the reported data on diagnoses and procedures translates directly to the funding of the hospitals. The data provide information from 1978 onwards. Hospitals mandatorily keep individual patient records related to hospitalisations and outpatient treatment for at least 10 years after the last record.

Cardiovascular events were defined as a composite of death from cardiovascular causes, myocardial infarction, stroke, amputation due to ischaemia and cardiac or peripheral revascularisation. If an event occurred as a direct consequence of, or as an adverse event related to another, immediately prior event (e.g. percutaneous transluminary coronary angioplasty immediately after acute myocardial infarction; see ESM section 2.1), this latter event was not considered in the analyses of separate endpoints. Self-reported cardiovascular events prior to randomisation were not considered. Microvascular complications were defined, measured and evaluated as described in ESM Section 3.

### Statistical analyses

All statistical analyses were conducted using the intention-to-treat principle. Adherence to the treatment was assessed at study visits in both groups based on interviews with patients.

Overall cumulative survival, as well as CVD-free survival, by time since randomisation was calculated for the two treatment groups by the Kaplan–Meier estimator. Median survival time from randomisation was compared between the treatment groups. CIs for the medians were calculated using bootstrapping. We specifically chose difference in median survival time between groups to make a clear statement in easily understandable terms in addition to the more extensively used, but more difficult to interpret, HR and RR reduction.

Proportional hazards Poisson models were fitted for mortality (both from cardiovascular causes and from other causes) and cardiovascular events, taking treatment group, age, sex and current CVD status into account, using smooth underlying hazards (time since baseline). Current CVD status was included in models for all-cause mortality as 0, 1, 2 or 3 or more cardiovascular events after randomisation. Thus, we modelled both death and (extra) CVD events as outcome, and the models were used to compute the average years of life lived and the years of life free of CVD during the study period and thereby the average number of years gained by intensive treatment. Models were checked for proportional hazards between randomisation groups by likelihood-ratio tests. Diabetes duration at baseline, as well as the interaction with treatment allocation, was included in a model to assess the effect of diabetes duration on mortality.

Except for end-stage renal disease (where exact date of transplantation or first dialysis treatment was known), the status of microvascular complications was only assessed at study visits, and the exact event date is therefore unknown. For analysis purposes a random date between the last day without and first day with the specified complication was imputed as the event date. When the progression between states jumped more than one category (e.g. from EURODIAB-score 2 to 5 between two observation points), random dates between the two observations for the steps were generated and used in analyses of transition rates. Sensitivity analyses were performed by repeating the random allocation of dates.

Transition rates between states and group comparison were analysed as for cardiovascular events. For each of the four types of outcome (retinopathy, autonomic neuropathy, peripheral neuropathy and albuminuria) we estimated the rates of transition between states of increasing severity and used these to construct plots showing the fraction of patients in each state at different times after randomisation.

A fuller account of statistical approaches and detailing of analyses can be found in ESM Sections 4–7. For the anthropometric, biochemical and physiological variables, the *t* test, Mann–Whitney *U* test and χ^2^ statistics were applied for the comparison of means, medians and proportions, respectively. In the results section all estimates are followed by 95% CIs in parentheses. Analyses were conducted using R, version 3.3.0, using the Epi package version 2.5 or Stata/IC version 14.1 for Windows (StataCorp LP, College Station, TX, USA).

## Results

Forty-two patients in the original intensive-therapy group and 24 in the original conventional-therapy group completed the entire follow-up period. Median observation time for patients completing follow-up was 21.2 years (range 20.2–21.9 years). Demographic, anthropometric, clinical variables and prescribed drug intake are presented in Table 1.

**Table 1:** Clinical, anthropometric and biochemical data and self-reported use of prescribed drugs

Variable	Baseline (1993)		End of intervention (2001)		End of follow-up (2014)	
	Intensive (*N* = 80)	Standard (*N* = 80)	Intensive (*N* = 67)	Standard (*N* = 63)	Intensive (*N* = 42)	Standard (*N* = 24)
Age, years	54.9 ± 7.2	55.2 ± 7.2	66.0 ± 7.0	66.1 ± 6.8	72.1 ± 6.4	71.9 ± 5.8
Age range, years	37–67	42–67	50–80	55–80	58–83	63–86
Proportion male sex, %	79	70	78	72	70	67
Diabetes duration, years	4 (0–30)	6 (0–29)	12 (7–28)	14 (7–37)	25 (20–41)	27 (21–51)
BMI, kg/m^2^						
Men	29.3 ± 3.6	30.3 ± 5.3	30.0 ± 4.3	30.8 ± 5.6	29.8 ± 4.4	31 ± 5.4
Women	31.1 ± 4.5	28.9 ± 3.8	33.8 ± 6.8	30.0 ± 4.4	31.5 ± 6.4	31.8 ± 3.8
Waist circumference, cm						
Men	105 ± 10	107 ± 14	108 ± 10	112 ± 14	109 ± 12	110 ± 15
Women	100 ± 14	101 ± 13	108 ± 14	107 ± 11	108 ± 11	112 ± 6
Blood pressure, mmHg						
Systolic	146 ± 11	149 ± 19	131 ± 13	146 ± 18*	145 ± 19	143 ± 18
Diastolic	85 ± 10	86 ± 11	73 ± 11	78 ± 10*	70 ± 10	68 ± 11
Fasting glucose, mmol/l	10.1 ± 3.1	10.5 ± 3.0	7.2 ± 2.5	9.9 ± 3.9*	7.7 ± 2.5	8.4 ± 2.7
HbA_1c_						
IFCC, mmol/mol	68 ± 6	73 ± 5	63 ± 10	75 ± 4*	58 ± 15	59 ± 12
DCCT, %	8.4 ± 2.7	8.8 ± 2.6	7.9 ± 3.1	9.0 ± 2.5	7.4 ± 1.4	7.5 ± 1.2
Stimulated C-peptide, pmol/l^a^	1438	1514	1140	1090	912	1059
Total cholesterol, mmol/l	5.4 ± 1.1	6.0 ± 1.3	4.1 ± 0.9	5.6 ± 1.3*	3.9 ± 0.9	4.1 ± 1.1
HDL-cholesterol, mmol/l	1.0 ± 0.2	1.0 ± 0.3	1.2 ± 0.4	1.2 ± 0.3	1.2 ± 0.5	1.1 ± 0.3
LDL-cholesterol, mmol/l	3.4 ± 0.9	3.5 ± 1.0	2.1 ± 0.8	3.3 ± 0.9*	2.1 ± 0.8	2.1 ± 0.8
Triacylglycerol, mmol/l	1.8 (1.3–2.7)	2.5 (1.4–3.2)	1.3 (0.9–2.3)	1.8 (1.3–3.2)*	1.2 (0.8–1.7)	1.4 (0.9–2.4)
p-Creatinine, μmol/l	78 ± 17	76 ± 16	102 ± 32	111 ± 85	108 ± 78	137 ± 119
u-AER, mg/24 h	78 (61–120)	69 (47–113)	46 (17–201)	126 (38–547)*	98 (38–309)	74 (18–377)
Self-reported drug intake (%)						
No glucose-lowering drugs	35	26	1	6	2	0
Metformin	13	19	50	34	59	50
Insulin	6	14	57	54	71	79
Any non-insulin hypoglycaemic	59	61	74	61	85	58*
Insulin + other hypoglycaemic drug	0	1	32	21	51	50
ACE inhibitor or ARB	20	19	97	70*	76	92
Diuretic	21	28	58	60	68	83
Calcium antagonist	14	6	36	29	51	46
Beta blocker	10	1	19	16	34	42
Other antihypertensive drug	1	1	4	6	24	25
Any antihypertensive drug	41	41	99	83*	93	100
Statin	0	3	85	22*	80	92
Fibrate	1	1	1	5	5	0
Antiplatelet therapy	16	15	87	56*	66	83

Data are means ± SD or medians (interquartile range) unless stated otherwise

^a^Median

*p < 0.05 for difference between groups

u-AER, urinary albumin excretion rate; ARB, angiotensin receptor blocker

### Mortality

**Fig. 2:**
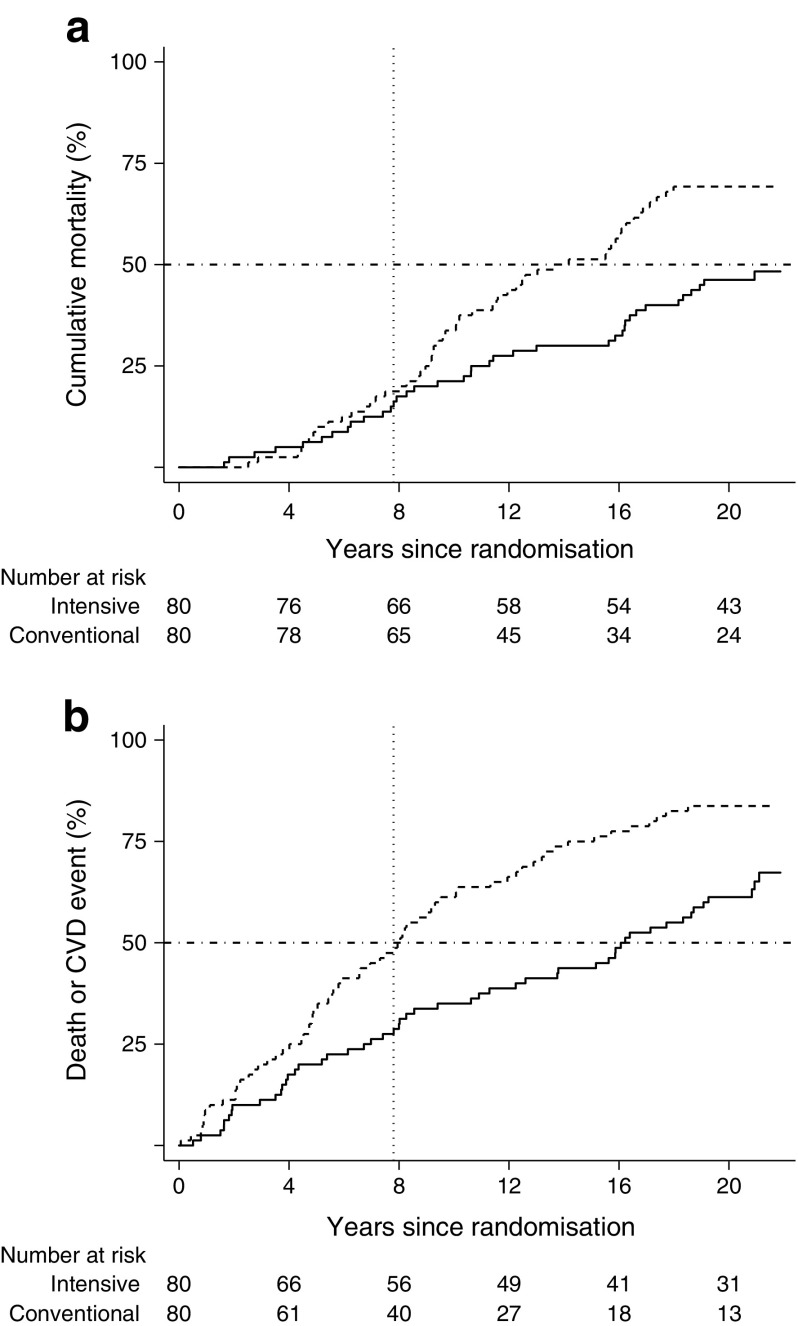
Cumulative mortality (**a**) and cumulative incidence of the composite cardiovascular or death endpoint (**b**). Solid lines, patients in the intensive-therapy group; dashed lines, patients in the conventional-therapy group; vertical dotted lines, end of trial and start of intensification of conventional-therapy group patients’ treatment; horizontal dashed lines intersect with survival curves at median survival time (**a**) and median CVD-free survival time (**b**). The median survival time in the original intensive-therapy group was at least 7.9 years longer than in the conventional-therapy group (48% of patients in the intensive-therapy group died during follow-up, so formally this might be an underestimate, since 50% mortality is required to calculate the median). The median difference in survival before first CVD event was 8.1 years in favour of the original intensive-therapy group

At the end of follow-up, 38 (48%) of the original intensive-therapy patients had died compared with 55 (69%) of conventional-therapy patients. Median survival time in the conventional-therapy group was 13.3 years, and the difference in median survival time after randomisation was therefore at least 7.9 years (95% CI 2.2, 9.6 years) (Fig. 2a). However, formally we were not able to calculate median survival time after randomisation in the intensive-therapy group (since 48% [and not 50% as required] of intensive-therapy group patients died before the end of follow-up) and the calculated differences in median survival might thus underestimate the real difference.

The observed median time to first CVD event or death was 8.0 years in the conventional-therapy group and 16.1 years in the intensive-therapy group; a difference of 8.1 years (95% CI 4.0, 12.6 years) (Fig. 2b).

**Fig. 3:**
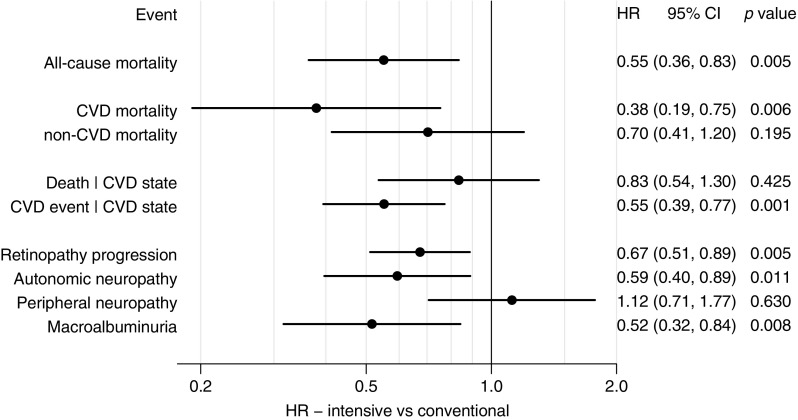
Forest plot of the HR (95% CI) for secondary and tertiary endpoints. Intensive vs conventional treatment. We found significant risk reductions for all-cause mortality, CVD mortality, CVD events and progression of retinopathy, autonomic neuropathy and macroalbuminuria. No difference was observed for non-CVD mortality, death after specific number of CVD events (Death | CVD state—i.e. no difference in mortality after first, second or third CVD event was observed between groups) and progression of peripheral neuropathy

HR (95% CI) estimates for secondary and tertiary endpoints are shown in Fig. 3. The overall, adjusted mortality rate in the original intensive-therapy group was reduced by 45% during the entire follow-up period. The absolute risk reduction in mortality in the intensive-therapy group was 21%.

Death from cardiovascular causes was reduced by 62% in the intensive-therapy group and no difference in non-cardiovascular mortality between groups was observed. No evidence that the mortality rate in the original conventional-therapy group increased after treatment intensification at 7.8 years was found (ESM Fig. 1); however, the CVD mortality rates were not proportional (the HR for intensive vs conventional were higher during the first 5 years after randomisation and lower later on). Following neutralisation of the intervention after 7.8 years, the rate-ratio for all-cause mortality was significantly in favour of the original intensive-therapy group.

We found a trend towards an effect of diabetes duration on mortality (HR 1.03 per year [95% CI 1.00, 1.07], *p* = 0.074; ESM Fig. 2). This association was equal for the two groups (*p* = 0.29).

### Cardiovascular disease

In the intensive-therapy group, 35 patients experienced a cardiovascular event compared with 51 patients in the conventional-therapy group, corresponding to a 51% RR reduction and 20% absolute risk reduction for incident CVD. Transitions between, and time in, different states of CVD (0, 1, 2 or 3 or more events since randomisation, respectively) are shown in ESM Fig. 5. Twenty-eight patients (35%) in the intensive group vs 13 (16%) in the conventional group completed the entire follow-up without any incident macrovascular events; HR for CVD event in the intensive-therapy group 0.55 (95% CI 0.39, 0.77; *p* < 0.001).

Patients in both groups with one post-baseline cardiovascular event had a higher mortality rate than patients without; HR 3.08 (95% CI 1.82, 5.19) and an almost linear increase in mortality of 2.08 (95% CI 1.73, 2.51) per extra event. A similar pattern was seen for further CVD events. When the hazard for mortality was adjusted for CVD status, there was no difference in mortality between groups (HR 0.83 [95% CI 0.54, 1.30], *p* = 0.43). Thus, the reduced mortality was primarily due to reduced risk of CVD.

**Fig. 4:**
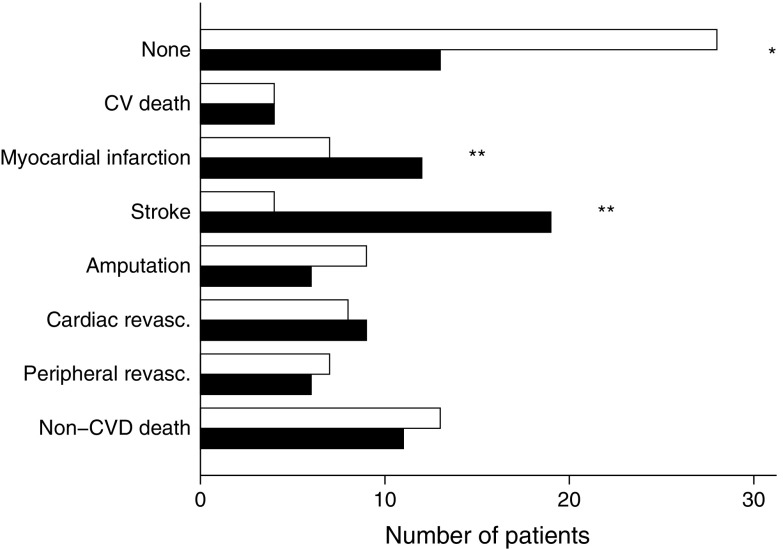
Distribution of type of first event by treatment allocation. White bars, intensive-therapy patients; black bars, conventional-therapy patients. *p < 0.05 and **p < 0.01 for difference between groups; revasc., revascularisation

The patients in the intensive group experienced a total of 90 cardiovascular events vs 195 events in the conventional group. Nineteen intensive-group patients (24%) vs 34 conventional-group patients (43%) experienced more than one cardiovascular event. No significant between-group difference in the distribution of specific cardiovascular first-event types was observed (Table 2 and Fig. 4).

**Table 2:** Distribution of specific cardiovascular event types by treatment allocation. Bottom row is the sum of the combined primary endpoint and other-cause mortality

Event type	Intensive-therapy group			Conventional-therapy group		
	No. of patients	No. of first events	No. of events	No. of patients	No. of first events	No. of events
Death from CVD	12	4	12	26	4	26
Death from other cause	26	13	26	29	11	29
Myocardial infarction	9	7	11	23	12	40
Stroke	10	4	11	25	19	41
Amputation	13	9	26	18	6	39
Cardiac revascularisation	10	8	17	20	9	29
Peripheral revascularisation	7	6	13	11	5	20
All non-fatal first events	35	34	78	52	52	169
Recurrent non-fatal events	19			34		
Combined primary endpoint	39	38	90	56	56	195
Total number of events		51	116		67	228

Bottom row is the sum of the combined primary endpoint and other-cause mortality

### Microvascular complications

Hazard rates of progression rates in microvascular complications compared with baseline status are shown Fig. 3. Sensitivity analyses showed a negligible effect of the random dates imputation.

**Fig. 5:**
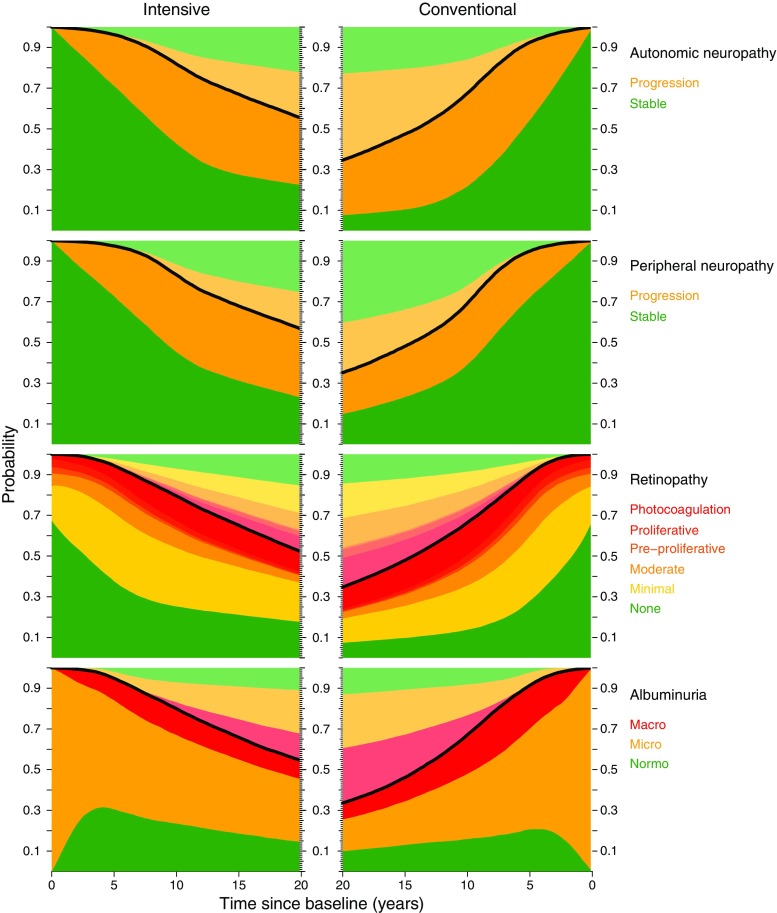
Progression of microvascular complications. The black line is the smoothed survival estimate. Green areas under the curves depict the probability of being alive without (progression in) the specified microvascular complication and the different shades of orange represent progression to the specified progression state after the given follow-up duration. The lightly coloured areas above the black curve depict the fraction of patients who died after progression corresponding to the specified disease states Both the ‘survival without progression’ area (dark green) and the total ‘survival’ area (area under the black curve) are significantly larger in the intensive-therapy group for autonomic neuropathy, retinopathy and nephropathy (albuminuria) (i.e. the risk of disease progression is decreased for these outcomes in the intensive-therapy group). No significant difference in the progression of peripheral neuropathy between groups was observed. An example of interpretation: for autonomic neuropathy, it is shown that the fraction of patients that died with no progression is similar in the two groups (light green), but the fraction of patients who died after progression (light orange) was significantly larger in the conventional-therapy group

Progression of retinopathy was decreased by 33% in the intensive-therapy group (Fig. 5). Blindness in at least one eye was reduced in the intensive-therapy group with an HR of 0.47 (95% CI 0.23, 0.98, *p* = 0.044). Autonomic neuropathy was decreased by 41% in the intensive-therapy group (Fig. 5). We observed no difference between groups in the progression of peripheral neuropathy (Fig. 5). Progression to diabetic nephropathy (macroalbuminuria) was reduced by 48% in the intensive-therapy group (Fig. 5). Ten patients in the conventional-therapy groups vs five patients in the intensive-therapy group progressed to end-stage renal disease (*p* = 0.061).

## Discussion

In the current report from the Steno-2 study we demonstrate that intensified treatment for 7.8 years was associated with a 7.9 years longer median lifespan over a period of 21.2 years follow-up. Furthermore, the increased lifespan was matched by the years gained free from incident CVD. The reduced mortality was caused by a decreased risk of incident CVD and cardiovascular mortality.

Absolute risk and RR reductions for all endpoints were well in line with earlier reported findings, confirming the durability of the intensified, multifactorial approach [13].

The frequency of recurrent events was high in both groups, but patients in the original conventional-therapy group experienced more than twice as many cardiovascular events per person than patients from the original intensive-therapy group. Only a few studies have reported results on recurrent events; none of these have been exclusively in patients with type 2 diabetes [2, 3] and the follow-up periods were much shorter, hence direct comparison is difficult.

In the Steno-2 study, we observed high rates of progression for microvascular complications with the vast majority of patients progressing in one or more complication types. Yet, we found significant and clinically relevant risk reductions for autonomic neuropathy, nephropathy, and retinopathy, as well as blindness, and a trend towards reduced risk for end-stage renal disease. Retinopathy grading was not corrected for cataract development and surgery, which made the longitudinal comparison less exact. For the rarer and later-onset complications (i.e. end-stage renal disease and blindness) competing risk from death might be a serious issue, underestimating the true effect of the intervention.

The risk reductions reported are high compared with those reported in single-risk-factor intervention trials [18–21]. Concomitant treatment of multiple risk factors seems to be of profound importance. A recent report from VADT (Veterans Affairs Diabetes Trial) demonstrated larger risk reductions in patients with diabetes mellitus achieving both HbA_1c_ and LDL-cholesterol goals compared with patients achieving only one of these goals [22]. Similar findings have been reported from the ADVANCE (Action in Diabetes and Vascular Disease: Preterax and Diamicron MR Controlled Evaluation) trial examining a combined approach of intensified blood pressure lowering and intensive glucose control [23] as well as from registry-based studies [2, 10]. In addition, analysis of the relation between number of risk factors simultaneously in control and cardiovascular outcomes in the BARI-2D (Bypass Angioplasty Revascularization Investigation 2 Diabetes) trial found a clear beneficial effect of having more risk factors in control [24], further supporting the concept of multifactorial treatment.

Long-term beneficial effects of glucose-lowering treatment in reducing microvascular complications have been demonstrated beyond the duration of a clinical trial in both type 1 and type 2 diabetes [20, 25, 26]. Lipid-lowering treatment has proven long-term beneficial effects with regards to CVD reduction [6]. However, the beneficial effects on cardiovascular outcomes of blood pressure- and glucose-lowering treatments seem to attenuate when treatment is relaxed post intervention [19, 21, 27]. Long-term beneficial effects in these trials have been termed ‘metabolic memory’ or ‘legacy effect’. In these trials, intensified intervention according to protocol was stopped at the end of the trial and the subsequent risk factor control was relaxed or not reported. In contrast, patients in the Steno-2 study’s intensive-therapy group continued treatment according to the original treatment goals while patients in the original conventional-therapy group started intensified treatment with identical targets during the follow-up period. Thus, from year 8 onwards all patients in both treatment arms in the Steno-2 study received identical treatment with similar post-trial risk factor levels in the two groups. We interpret the continuing beneficial effects seen in the trial as a direct consequence of early intervention intensification in patients at lower absolute risk for late diabetic complications compared with a situation wherein increased vascular damage is already present with intensification in later stages of the disease. This concept of early intervention in patients at lower risk has just proven beneficial for combined treatment of lipid and blood pressure lowering in patients at intermediate risk of, but without, CVD [28].

The Steno-2 study is robust in the sense that data on endpoints are considered complete due to the quality of relevant databases combined with low dropout rate and endpoint adjudication by an external expert committee blinded for treatment allocation. Additionally, the study design resembles a real-life situation, where researchers did not have direct influence on medicine compliance or lifestyle.

In conclusion, we found that intensified, multifactorial treatment of type 2 diabetes with microalbuminuria for 7.8 years compared with conventional treatment increases median life length by 7.9 years over 21.2 years of follow-up, and that these gained years were matched by years free from cardiovascular complications.

We must emphasise the significance of early, intensified risk factor control in patients with complicated type 2 diabetes. This approach is already broadly implemented according to clinical guidelines and the present findings should lead to even more focus on the potential preventive effects.

## Electronic supplementary material

Below is the link to the electronic supplementary material.

ESM (PDF 758 kb)

## Abbreviation

CVD: Cardiovascular disease
