# Supplementary material for: Years of life gained by multifactorial intervention in patients with type 2 diabetes mellitus and microalbuminuria: 21 years follow-up on the Steno-2 randomised trial
Source: Diabetologia. 2016 Aug 16;59(11):2298–307. doi: 10.1007/s00125-016-4065-6 (PMC5506099; doi:10.1007/s00125-016-4065-6)
Supplement: Supplementary file 1 — (PDF 758 kb) [file 125_2016_4065_MOESM1_ESM.pdf]

Years of life gained by multifactorial intervention in patients with type 2 diabetes mellitus and microalbuminuria — 21 years follow up on the Steno-2 randomized trial

Electronic Supplementary Material

SDC

June 2016
Final version

Compiled Wednesday 29th June, 2016, 13:09
from: /home/bendix/sdc/coll/jchq/r/ESM.tex

Bendix Carstensen Steno Diabetes Center, Gentofte, Denmark
& Department of Biostatistics, University of Copenhagen
bxc@steno.dk
http://BendixCarstensen.com

Contents

1 Treatment targets 0

2 Definitions of endpoints used in the Steno-2 study 1
2.1 Method to determine exclusion of recurrent events . . . 4

3 Microvascular outcome assessments 6

4 Statistical analyses 7
4.1 Median survival . . . 7
4.2 Poisson modeling . . . 7
4.3 Cumulative risk . . . 7
4.3.1 Practical implementation . . . 7

5 Mortality 8
5.1 Analysis of rates . . . 8
5.2 Diabetes duration . . . 8
5.3 Survival curves . . . 8

6 CVD events and death 11

7 Microvascular events at clinical visits 16
7.1 Retinopathy . . . 17
7.2 Neuropathy . . . 21
7.2.1 Autonomic neuropathy . . . 22
7.2.2 Peripheral neuropathy . . . 23
7.3 Albuminuria . . . 24

References 26

List of Tables

1 Treatment targets for patients in the two treatment groups. 0
2 HRs of overall and CVD / other mortality; intensive versus conventional. . . 8
3 Intensive vs. Conventional HRs of CVD event and death. 12
4 Expected lifetime in the Steno 2 cohort. 12
5 Expected lifetime in the Steno 2 cohort by sex and age. 13
6 HRs of retinopathy improvement, progression and death between intensive and conventional groups. All HRs are controlled for attained retinopathy state. 17

List of Figures

1 Overall mortality rates and hazard ratio in the two groups. 9
2 Overall mortality HR by duration of diabetes. 10
3 Survival curves for Steno 2 patients by sex and age. 10
4 Transitions between states of CVD and death. 11
5 CVD state distribution by time since baseline. 13
6 CVD state distribution by time since baseline, subdivided by sex and age. . . 14
7 Cumulative risks of death and CVD. 15
8 Transitions between states of retinopathy and death, allowing improvements in retinopathy status 18
9 Transitions between states of retinopathy and death, allowing only deterioration in retinopathy status. 19
10 Distribution of retinopathy, allowing retinopathy improvement. 20
11 Distribution of retinopathy, dis-allowing retinopathy improvement. 20
12 Transitions between states of neuropathy progression. 21
13 Fraction of patients with progression of autonomic neuropathy. 22
14 Fraction of patients with progression of peripheral neuropathy. 23
15 Transitions between states of albuminuria. 24
16 Intervention effects for transitions between albuminuria states. 25
17 Fraction of patients in different states of albuminuria. 25

1 Treatment targets

ESM Table 1: *Treatment targets for the patients in the two treatment groups. Patients in the conventional therapy group received treatment according to existing Danish guidelines, which were updated in 2000.*

	Conventional Therapy		Intensive Therapy	
	1993–1999	2000–2001	1993–1999	2000–2001
Systolic blood pressure (mm Hg)	< 160	< 135	< 140	< 130
Diastolic blood pressure (mm Hg)	< 95	< 85	< 85	< 80
HbA1c (%)	< 7.5	< 6.5	< 6.5	< 6.5
Fasting serum total cholesterol (mmol/L)	< 6.5	< 4.9	< 4.9	< 4.5
Fasting serum triglycerides (mmol/L)	< 2.2	< 2.0	< 1.7	< 1.7
Treatment with ACE-inhibitor irrespective of blood pressure	No	Yes	Yes	Yes
Aspirin Therapy:				
Known ischemia	Yes	Yes	Yes	Yes
Peripheral vascular disease	No	No	Yes	Yes
No known CHD or peripheral vascular disease	No	No	No	Yes

2 Definitions of endpoints used in the Steno-2 study

An independent, masked endpoint committee consisting of two specialists in cardiology and one specialist in diabetology evaluated all cases and classified cardiovascular events into the following categories:

1.0 Cardiovascular death

- 1.1 Sudden death: Sudden death presumed to be due to ischemic cardiovascular disease, occurring within 24 hours of the onset of symptoms without confirmation of cardiovascular disease, and without clinical or post mortem evidence of other etiology.
- 1.2 Fatal myocardial infarction: death within 7 days of the onset of documented myocardial infarction (see 2.0).
- 1.3 Congestive heart failure: death due to clinical, radiological or post mortem evidence of congestive heart failure without clinical or post mortem evidence of an acute ischemic event (which should then be coded as the cause). Cardiogenic shock to be included.
- 1.4 Post cardiovascular invasive interventions: death associated with the intervention: within 30 days of cardiovascular surgery, or within 7 days of cardiac catheterization, or angioplasty, atherectomy, stent deployment or other invasive coronary or peripheral vascular interventions.
- 1.5 Documented arrhythmia: death due to bradyarrhythmias or tachyarrhythmias not induced by an acute ischemic heart disease event (which should then be coded as the cause).
- 1.6 Death following non-cardiovascular surgery: death due to cardiovascular causes as defined in 1.1-1.5 and 1.7-1.8 and within 30 days of surgery.
- 1.7 Fatal stroke: death due to stroke occurring within 7 days of the signs and symptoms of a stroke.
- 1.8 Other cardiovascular diseases: death due to other vascular diseases including pulmonary emboli, and abdominal aortic aneurysm rupture.
- 1.9 Presumed cardiovascular death: suspicion of cardiovascular death with clinically supporting evidence which may not fulfill criteria otherwise stated. Example: Patient admitted with typical chest pain of 3 hours duration and treated as a myocardial infarction, but without ECG and enzymatic documentation to meet normal criteria.

2.0 Myocardial infarction (MI)

- 2.1 Q-wave MI: in comparison to the last ECG, presence of at least one new significant Q-wave on the standard 12-lead ECG as described in the Minnesota Code, and at least one of:
  1. Typical symptoms (e.g. typical ischemic chest pain lasting more than 30 minutes and/or

2. Significant elevation of serum enzymes — presence of any of the following criteria:
   a) elevation of troponin to above the upper limit of normal for the laboratory that performed the test
   b) elevation of creatin-kinase MB (CK-MB) to twice the upper limit of normal for the laboratory that performed the test
   c) elevation of total CK to at least twice the upper limit of normal for the laboratory that performed the test
   d) Elevation of aspartate aminotransferase (ASAT), alanine aminotransferase (ALAT), lactate dehydrogenase (LDH) to at least twice the upper limit of normal for the laboratory that performed the test with a characteristic pattern.

2.2 Non Q-wave MI: defined as a significant elevation of cardiac enzymes (at least twice the upper limit of normal) with or without characteristic pain in absence of new significant Q-wave.

2.3 Probable non Q-wave MI: presence of new and persistent ST-T changes (more than 24 hours in duration) on the ECG with characteristic symptoms of ischemic chest pain with- out documentation of enzyme elevation.

2.4 Silent MI: development of new significant Q-waves on the ECG (Minnesota Code) in at least two adjacent leads in the absence of any other evidence of myocardial infarction (in this case the date of event will be assessed as halfway between the date of discovery and last normal ECG).

2.5 Non-fatal MI post cardiovascular invasive interventions: MI (as defined in 2.1, 2.2, 2.3 or 2.4) associated with the intervention within 30 days of cardiovascular surgery, or within 7 days of cardiac catheterization, or angioplasty, atherectomy, stent deployment or other invasive coronary or peripheral vascular interventions.

2.6 Non-fatal MI post non-cardiovascular surgery: MI (as defined in 2.1, 2.2, 2.3 or 2.4) occurring within 30 days of non-cardiovascular surgery.

3.0 Stroke

3.1 Definite ischemic stroke: a CT or MRI scan within 2 weeks of onset of a definite stroke (focal neurological deficit greater than 24 hours) with evidence of infarction, or autopsy confirmation.

3.2 Definite hemorrhagic stroke (primary intracerebral, sub- arachnoid, or secondary to cerebral infarction): confirmation with a CT or MRI scan within 2 weeks of stroke, or at autopsy or by lumbar puncture.

3.3 Stroke of unknown etiology: definite stroke of unknown etiology when CT, MRI or autopsy are not done, or where CT or MRI scan does not reveal pathology.

3.4 Non-fatal stroke post cardiovascular invasive interventions: stroke (as defined in 3.1, 3.2 or 3.3) associated to the intervention within 30 days of cardiovascular surgery, or within 7 days of cardiac catheterization, or angioplasty, atherectomy, stent deployment or other invasive coronary or peripheral vascular interventions.

- 3.5 Non-fatal stroke post non-cardiovascular surgery: stroke (as defined in 3.1, 3.2 or 3.3) occurring within 30 days of non- cardiovascular surgery.

4.0 Transient ischaemic attacks (TIA)

- 4.1 Definite TIA: focal neurological deficits with duration of less than 24 hours. Deficits must be observed and described by a physician.
- 4.2 Probable TIA: focal neurological deficits with duration of less than 24 hours. Deficits not observed or described by a physician.
- 4.3 TIA post cardiovascular invasive interventions: TIA (as defined in 4.1 or 4.2) associated to the intervention within 30 days of cardiovascular surgery, or within 7 days of cardiac catheterization, or angioplasty, atherectomy, stent deployment or other invasive coronary or peripheral vascular interventions.
- 4.4 TIA post non-cardiovascular surgery: TIA (as defined in 4.1 or 4.2) occurring within 30 days of non-cardiovascular surgery.

5.0 Amputation (includes both amputation and exarticulation)

- 5.1 Amputation caused by cardiovascular disease
- 5.2 Amputation caused by pressure wound/gangrene/infection

6.0 Invasive cardiovascular procedures

- 6.1 Coronary artery bypass graft (CABG)
- 6.2 Percutaneous transluminal coronary angioplasty (PTCA)
- 6.3 Attempted PTCA (to be used in case of an unsuccessful attempt)
- 6.4 Coronary stent deployment
- 6.5 Coronary arteriography with stenosis

7.0 Peripheral vascular procedures

- 7.1 Bypass surgery; state localization:
  - 7.1.1 lower extremity
  - 7.1.2 upper extremity
  - 7.1.3 carotid
- 7.2 Percutaneous transluminal angioplasty (PTA); state localization:
  - 7.2.1 lower extremity
  - 7.2.2 upper extremity
  - 7.2.3 carotid
- 7.3 Attempted PTA (to be used in case of an unsuccessful attempt); state localization
  - 7.3.1 lower extremity
  - 7.3.2 upper extremity

        - 7.3.3 carotid
    - 7.4 Stent deployment; state localization:
        - 7.4.1 lower extremity
        - 7.4.2 upper extremity
        - 7.4.3 carotid
    - 7.5 Thrombendarterectomy/thrombectomy; state localization:
        - 7.5.1 lower extremity
        - 7.5.2 upper extremity
        - 7.5.3 carotid
    - 7.6 Stenosis in a. carotis verified by arteriography or Doppler ultrasound

- 8.0 Death from other than cardiovascular cause
    - 8.1 cancer
    - 8.2 suicide
    - 8.3 hypoglycemia
    - 8.4 accident
    - 8.5 unspecified

- 9.0 Ischaemia in ECG
    - 9.1 Ischemia in resting ECG: Minnesota code 1.1–1.3, 4.1–4.4, 5.1–5.8 or 7.1
    - 9.2 Ischemia in work load ECG: ST-depression of more than 1 mm in any lead

- 10.0 Significant decline in distal blood pressure gradient: decline in systolic blood pressure gradient of at least 28 mm Hg between the right arm and great toe in one or both legs.

2.1 Method to determine exclusion of recurrent events

In the analyses of recurrent event rates, events directly related are a confounding factor. An example hereof is the patients having a myocardial infarction and being revascularized at the hospital within hours to days of the MI.

Recurrent CVD events are excluded from the recurrent event analyses if they occur as a secondary event within 30 days from a primary event and fulfill the below stated criteria i.e. are directly derived from those events.

The event codes (#x.) refer to the codes in the previous section (2).

a) Myocardial Infarction (#2.): Excluded if the primary event is MI (#2.) or invasive cardiovascular procedures (#6.).

b) Stroke (#3.): Excluded if the clinical manifestations or documented affected vascular bed is similar to primary event.

c) Amputation (#5.): Excluded if the primary event is amputation more distally on the same limb or if vascular procedure on same limb (#7.1-7.5).

d) Invasive cardiovascular procedure (IVP) (#6.): Excluded if primary event is MI (#2.) or IVP (#6.).

e) Peripheral vascular procedures (PVP) (#7.): Excluded if primary event is PVP (#7.) on same vascular bed. Carotid Stenosis (#7.6) is excluded if primary event is stroke (#3. or #4.)

f) Dead from CVD (#1.) and death from other causes (#8.) are never excluded

g) Ischemia in ECG (#9) and distal blood pressure gradient (#10) are not considered at all with regards to cardiovascular events in the follow-up.

In cases where a secondary event is excluded from the recurrent event analyses any tertiary event is treated as the recurrent event. If the tertiary event fulfills the above criteria too, any fourth event is regarded as the recurrent event etc.

3 Microvascular outcome assessments

Diabetic nephropathy was defined as urinary albumin excretion rate exceeding 300 mg per 24 hours in two of three consecutive sterile urine specimens measured at the study visits.

Diabetic retinopathy was graded according to the six-level grading scale of the European Community-funded Concerted Action Programme into the Epidemiology and Prevention of Diabetes (EURODIAB) by two independent eye specialists who were unaware of the patients' treatment allocation. Progression of retinopathy was defined as an increase of at least one level in the EURODIAB grading scale in either eye. Retinopathy was not adjusted for cataract.

Blindness was defined according to the criteria of the World Health Organization as a maximally corrected visual acuity of less than 6/60 in either eye (less than 20/200 on the Snellen visual-acuity scale).

Peripheral neuropathy was measured with a biothesiometer and graded according to age by a nomogram.

The diagnosis of autonomic neuropathy was based on a measurement of the changes in RR interval on an electrocardiogram obtained during paced breathing and an orthostatic hypotension test. Less than four milliseconds variation in RR-interval was coded as abolished, four to six milliseconds as impaired and more than six milliseconds as normal.

A drop in systolic blood pressure of 25 mmHg or more or if the patient experienced dizziness was regarded as a positive orthostatic hypotension test.

4 Statistical analyses

4.1 Median survival

Median survival in each of the intervention groups was estimated from Kaplan-Meier curves for the two outcomes (mortality and mortality / CVD event) and deriving the median survival and the difference in these. Confidence intervals for the difference in median survival were derived by bootstrapping (resampling *with* replacement) and taking the 2.5, 50 and 97.5 percentiles of the realized differences in median survival from 5,000 analyses of bootstrap samples after imputing 21 years when no median was found.

4.2 Poisson modeling

Analyses of survival (time-to-event) data were performed with Poisson modeling of data sets where follow-up was split in 1-month intervals and the baseline hazard modeled by a cubic spline. This type of modeling gives estimates for regression parameters that are practically indistinguishable from those from the corresponding Cox-model, but in addition provides direct access to an estimate of the baseline hazard which is complicated to extract from a Cox model.

The models also produce survival functions that are continuous functions of time.

Furthermore tests for proportionality of hazards along the time scale are very simple; they are merely simple interaction tests.

4.3 Cumulative risk

For all outcomes we computed the cumulative risk, which for non-fatal outcomes depends not only on the rate of the event in question, but also on the mortality rate. If more than one outcome (such as different degrees of retinopathy) is of interest, the probability of a given type of event depends on event rates of all types of events. Analytic expressions of these probabilities are largely intractable, and hence simulation from a complete model for all event and mortality rates (multistate model) is the only feasible approach to determine these probabilities.

Poisson models lend themselves particularly easily to this type of simulation of outcomes in multistate models.

4.3.1 Practical implementation

All results here are based on a complete report of all analyses available as `http://bendixcarstensen.com/St2/steno2.pdf`.

The follow-up in terms of risk time and events for any given type of events have been set up in **R**(version 3.3.0 [1]), using `Lexis` objects [2, 3] as implemented the `Epi` package (version 2.5 [4]).

Transitions and follow-up were displayed in diagrams to give an overall impression of the course of events in the two allocation groups. Poisson modeling based on this set up were used as input to the `simLexis` function that will simulate the life-course of a population according to a set of specified transition rates. This was used to derive cumulative risks of specific events.

5 Mortality

5.1 Analysis of rates

Total mortality as well as cause-specific mortality (CVD / other) was analyzed by proportional hazards Poisson-models with effects of time from baseline (as a natural spline), allocation group, age at baseline and sex. Proportional hazards were tested by including an interaction between intervention and time since baseline.

In table 2 is shown the HRs comparing the intensive with the conventional arm, both with and without control for age and sex.

ESM Table 2: *HRs of overall and CVD / other mortality; intensive versus conventional.*

				Cause of death					
	All causes			CVD			non-CVD		
	HR	95% CI	P	HR	95% CI	P	HR	95% CI	P
Overall	0.55	(0.36;0.83)	0.005	0.38	(0.19;0.75)	0.006	0.70	(0.41;1.20)	0.195
w/ age, sex	0.53	(0.35;0.80)	0.003	0.35	(0.18;0.71)	0.003	0.69	(0.40;1.17)	0.168

Overall and CVD mortality rates by time since baseline are shown for the two randomization groups in figure 1. It is seen that mortality is small in the beginning of the trial but increasing and then flattening off after some 8 years of follow-up.

There were no indication of non-proportional hazards for all-cause mortality, p=0.447; but for CVD mortality there was, p=0.040.

The CVD mortality rates are shown by time in figure 1, the non-proportionality of hazards (non-constant HR between intensive and conventional) is evident, where the intensive vs conventional HR is smaller, primarily *after* the intervention period.

5.2 Diabetes duration

We explored if diabetes duration at baseline influenced the mortality; we found a borderline significant effect of diabetes duration at base on overall mortality; an increase of 3.4% per year of diabetes duration (95% CI: −0.3;7.2).

There were no significantly different effect of diabetes duration between the two intervention arms, p=0.287 for linear interaction and p=0.769 for quadratic. There was a weak indication that the effect of intervention was largest for those with a short duration of diabetes (Figure 2).

5.3 Survival curves

Based on the non-proportional hazards Poisson model including age at baseline and sex, we derived survival curves for men, resp women entering the study at ages 45, 50,...,65 (approximately quartiles of age); shown in figure 3.

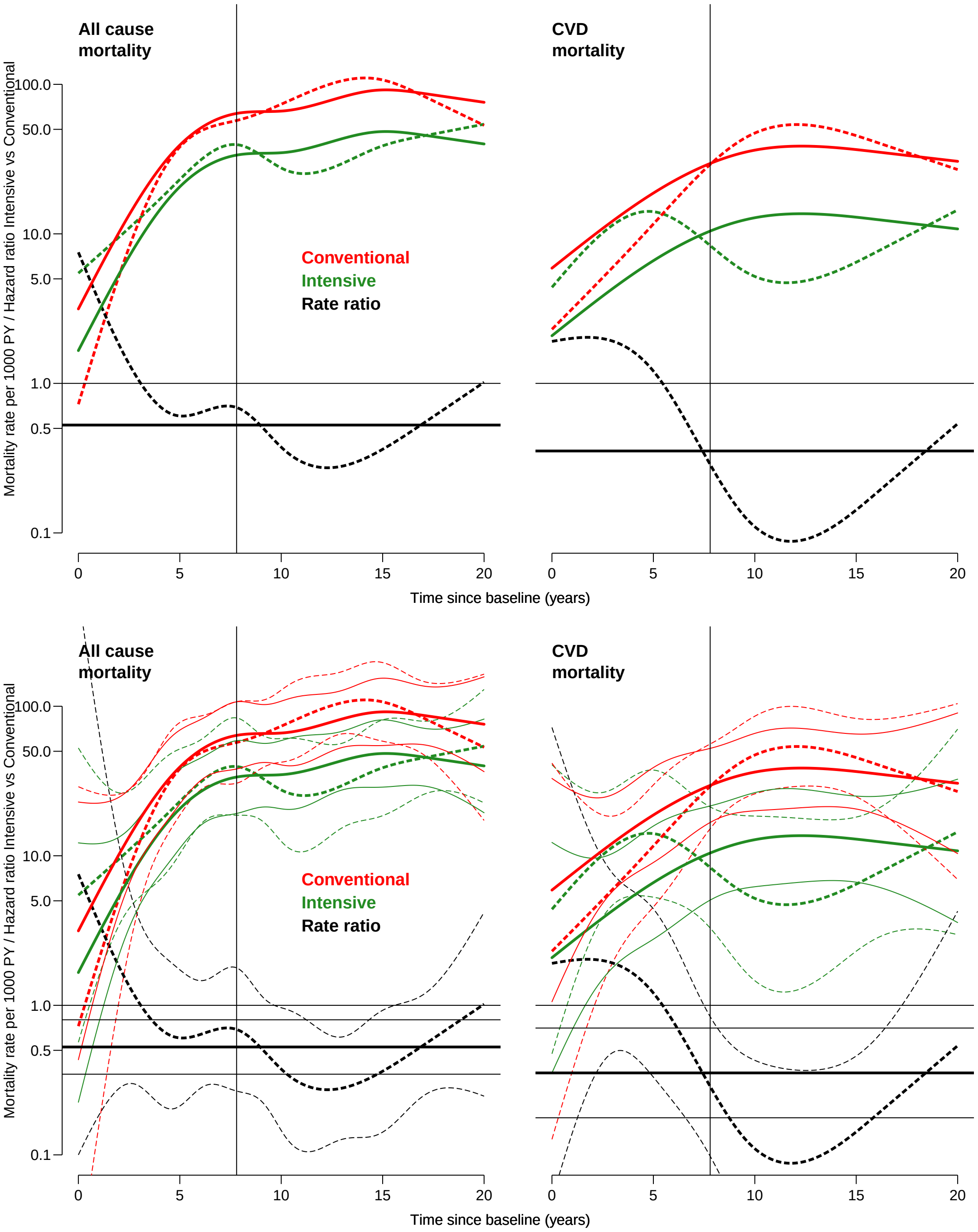

ESM Figure 1: *Overall mortality rates and hazard ratio in the two groups for all-cause mortality (left) and CVD mortality (right). The full lines are hazards and HRs assuming proportional hazards, broken lines are the hazards without the proportional hazards assumption. The vertical line indicates the intervention end (start of intensification in the conventional group); there were no indication of change in HR at this point.*
The rates are from models that include sex and age at baseline as linear terms on the log-mortality scale, and mortality rates are shown for a man aged 55 at baseline. The bottom panels are the same curves amended with 95% confidence intervals.

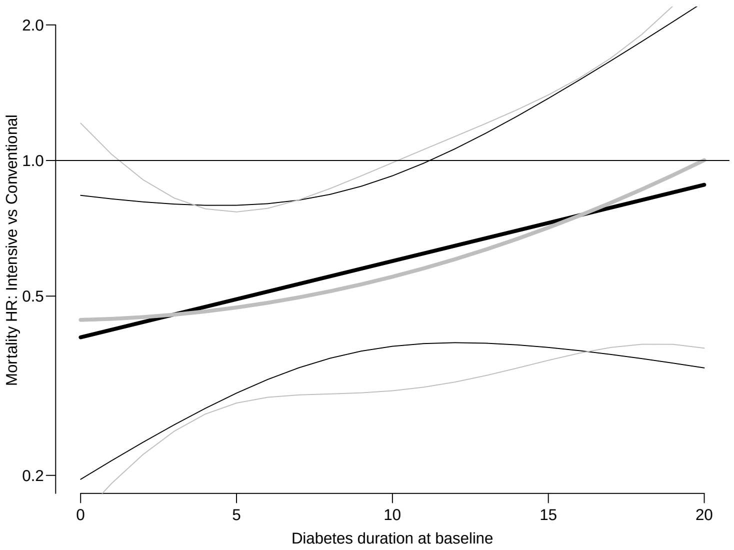

ESM Figure 2: *Mortality hazard ratio between intensive and conventional by diabetes duration at baseline, estimated from two different interaction models, one with log-linear effects (black) and another with log-quadratic effects (gray) of DM duration on HR. Neither of the interactions are statistically significant, p=0.287, p=0.769 respectively.*

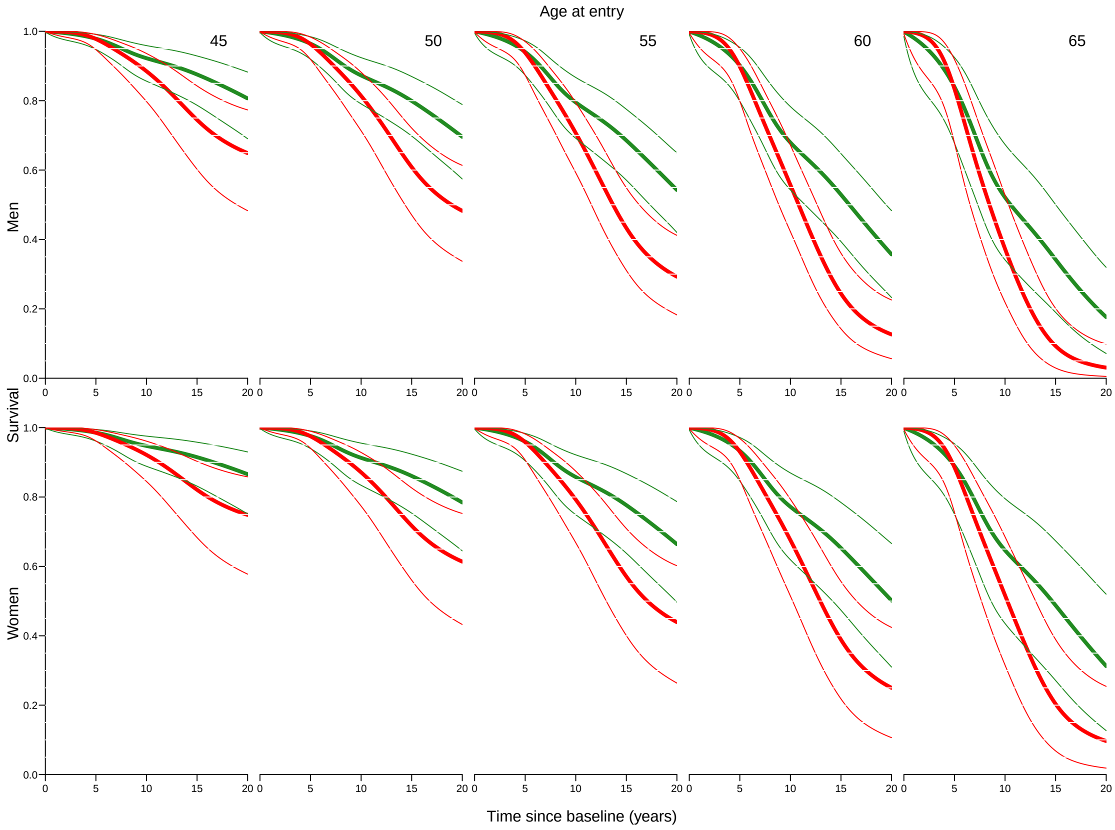

ESM Figure 3: *Survival curves for the Steno 2 patients entering at different ages, separately for each sex. Based on a non-proportional hazards model with log-linear effect of sex and age at entry. Green: intensive group, red: conventional.*

6 CVD events and death

Dates of cardiovascular events post baseline were used for evaluation of CVD morbidity — up to 3 was counted. We modeled transition rates between CVD states (0,1,2,3+ events) and mortality rates separately (figure 4). The total model for all transitions shown was used to estimate the fractions of the population that had 0,1,2 and 3+ CVD events at any one time, as well as the expected lifetime with and without CVD during the first 20 years.

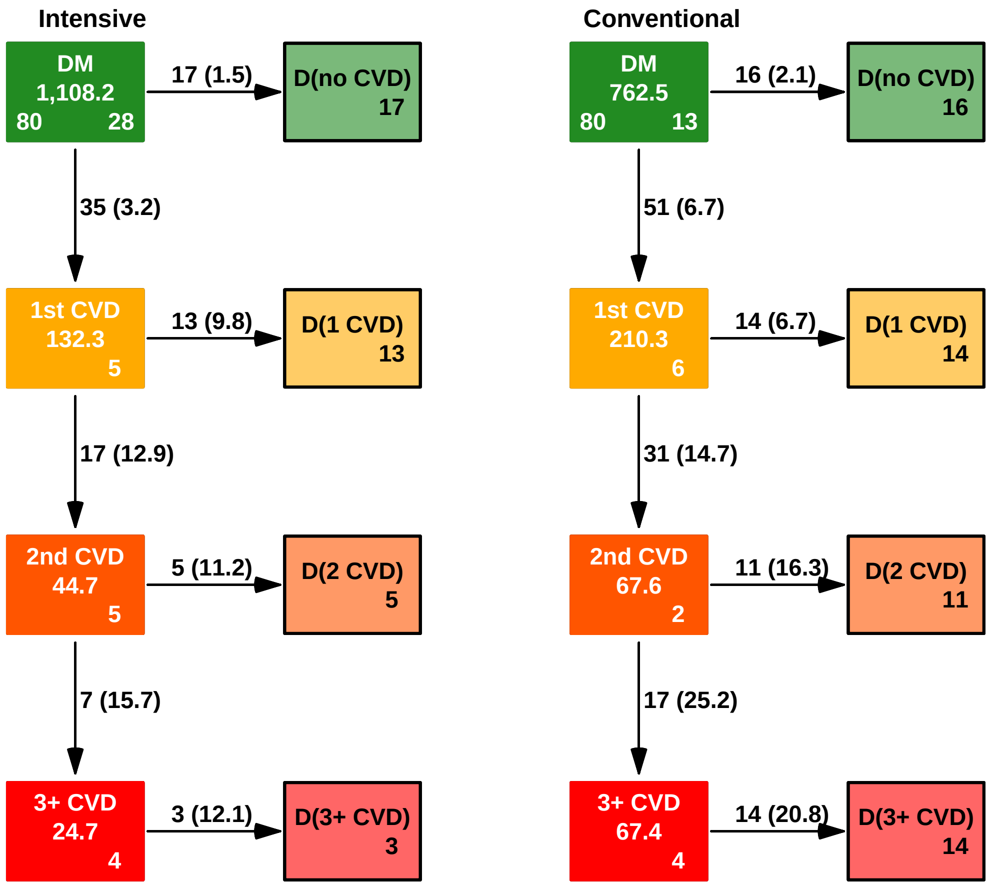

ESM Figure 4: *Transitions between states of CVD and death. The number in the center of the boxes is the person-years (PY), the numbers at the bottom is the number of patients starting, resp. ending their follow-up in each box (state), and the numbers on the arrows are the number of transitions and the overall transition rates per 100 PY.*

The intensive versus conventional HR was constant across states of CVD event both for occurrence of (extra) CVD events (p=0.261) and mortality (p=0.438). The HR between the intensive and the conventional was 0.55 (0.39;0.77) for CVD event and 0.83 (0.54;1.30) for mortality, see table 3. Thus the mortality for a given CVD state is not significantly different between the two groups, but the CVD progression is. Since CVD progression is associated with strongly increasing mortality (table 3), there is a significant overall mortality difference between intervention groups, which is clearly mediated by smaller

ESM Table 3: *Intensive vs. Conventional HRs of CVD event and mortality. The HRs are assumed constant across CVD states. The baseline mortality and CVD event rates depend on current CVD status, but in the same way for both intervention groups. 95% CI are given in parenthesis.*

	Mortality	CVD event
HR, Intensive vs. Conventional	0.83 (0.54; 1.30)	0.55 (0.39;0.77)
H_0: PH btw. CVD groups	p=0.438	p=0.261
H_0: HR = 1	p=0.425	p=0.001
HR vs. 0 CVD events:		
0 (ref.)	1.00	1.00
1	3.08 (1.82; 5.19)	2.43 (1.67;3.52)
2	4.42 (2.36; 8.29)	3.48 (2.15;5.64)
3+	7.76 (4.11;14.65)	
HR per event	2.08 (1.73; 2.51)	2.17 (1.75;2.68)

CVD progression rates in the intensive group.

The probability of having 0, 1, 2 and 3+ CVD events at different times after baseline is shown in figure 5 for the Steno 2 patients. In figure 6 is shown the same but for patients of specific sex and age at baseline.

From these figures it is also possible to derive the cumulative risk of death and CVD by intervention, sex and age at entry, and thus quantify the intervention effect in terms of the age effect on these quantities. From figure 7 we see that the intervention effect on overall cumulative mortality corresponds to an age-difference of some 5 years, whereas the effect on cumulative risk of CVD, the intervention brings the cumulative risk of CVD among 65 year old (at entry) below the level of the conventionally treated 45 year old.

The area of the colored areas in figure 5 represent the expected time spent alive with and without CVD during the first 20 years after baseline, these and the difference between treatment groups) are shown in table 4 averaged over the entire Steno 2 population, and in table 5 by age and sex. It is seen that the years of life gained is about 1.5, but the years without CVD gained is 2.6.

ESM Table 4: *Expected lifetime (years) in the Steno 2 cohort during the first 20 years after baseline by treatment group and CVD status.*

State	Intensive	Conventional	Int.−Conv.
Alive	15.6	14.1	1.5
No CVD	12.7	10.0	2.6
Any CVD	3.0	4.1	−1.1

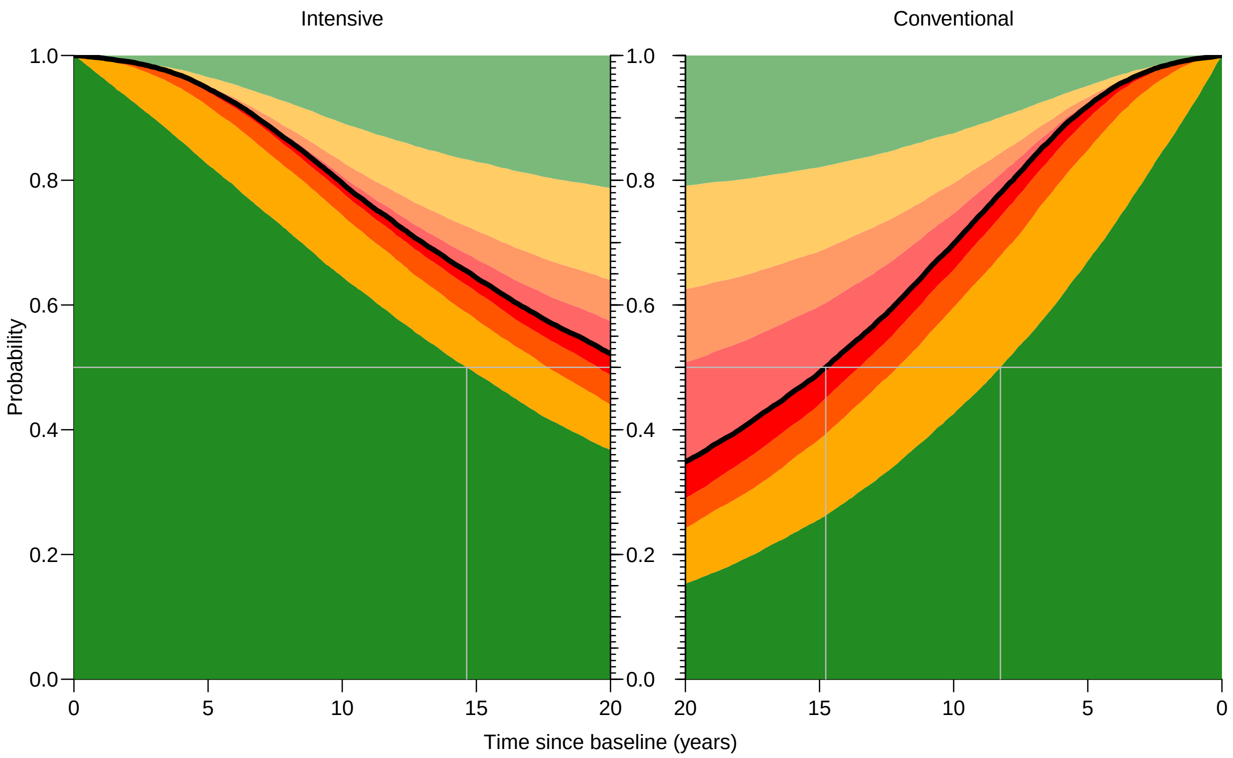

ESM Figure 5: *State distribution by time since baseline for a patient population with the same age- and sex-distribution as the Steno 2 patients. The thin gray lines indicate the median survival resp. CVD-free survival. The colors of the states correspond to the coloring in figure 4. The size of each coloured area corresponds to the expected time spent in the corresponding state during the first 20 years of follow-up; these are summarized in tables 4 and 5.*

ESM Table 5: *Expected lifetime (years) during the first 20 years after baseline by sex, age, treatment group and CVD status.*

sex		Men			Women		
state	age	Int.	Conv.	Int.−Conv.	Int.	Conv.	Int.−Conv.
Alive	45	18.5	17.5	1.0	19.1	18.4	0.7
	50	17.2	16.1	1.1	18.0	17.2	0.8
	55	15.6	13.8	1.8	17.4	15.9	1.6
	60	13.9	11.6	2.2	15.5	13.7	1.8
	65	11.2	9.5	1.8	13.3	11.4	2.0
No CVD	45	14.9	12.5	2.4	15.8	14.3	1.5
	50	14.0	11.1	2.9	15.1	12.9	2.2
	55	12.2	9.7	2.5	14.3	11.6	2.7
	60	10.9	8.2	2.7	12.4	9.9	2.6
	65	9.0	6.7	2.2	10.7	8.3	2.4

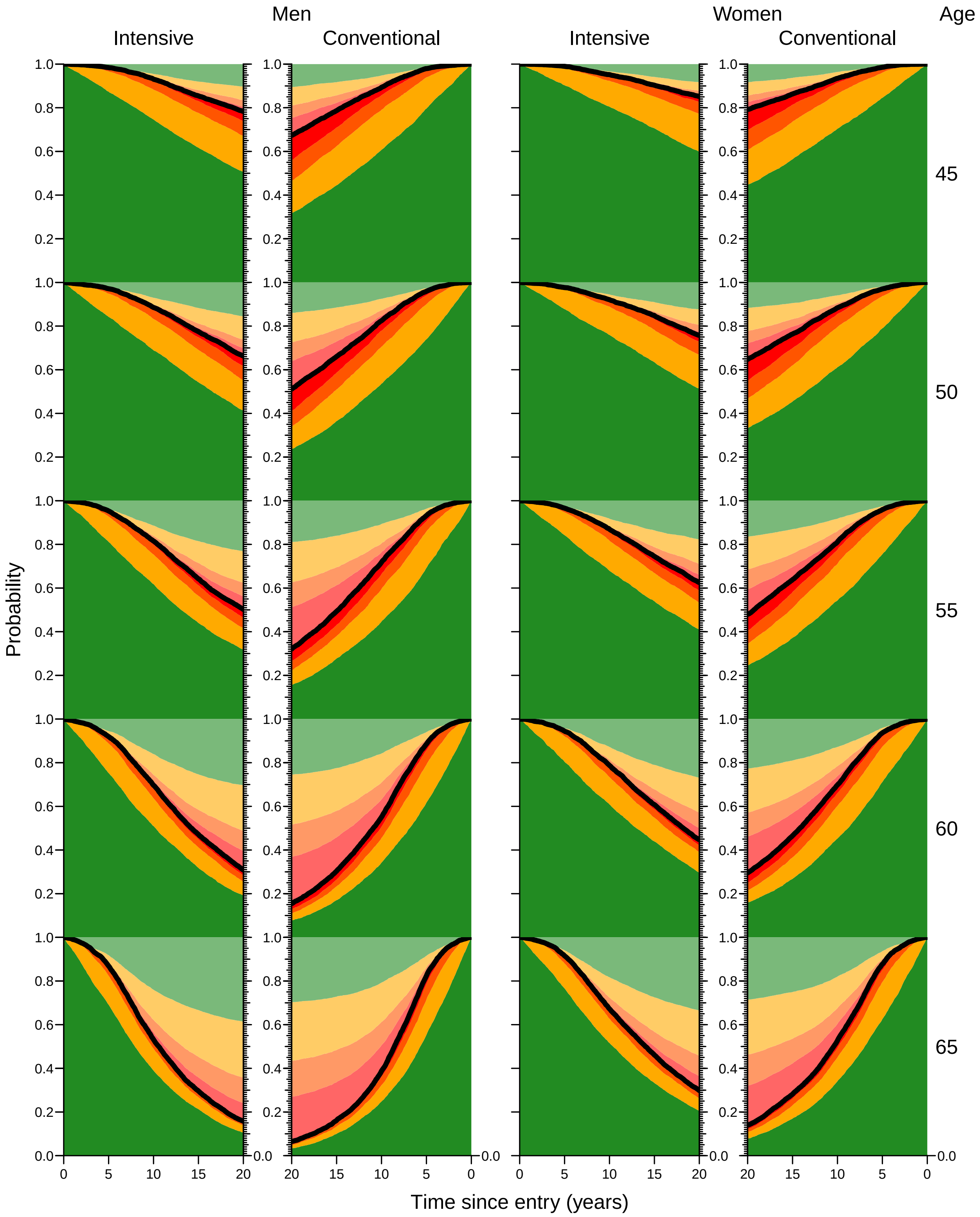

ESM Figure 6: *CVD state distribution by time since baseline, subdivided by sex and age at baseline. The colors of the states correspond to the coloring in figure 4.*

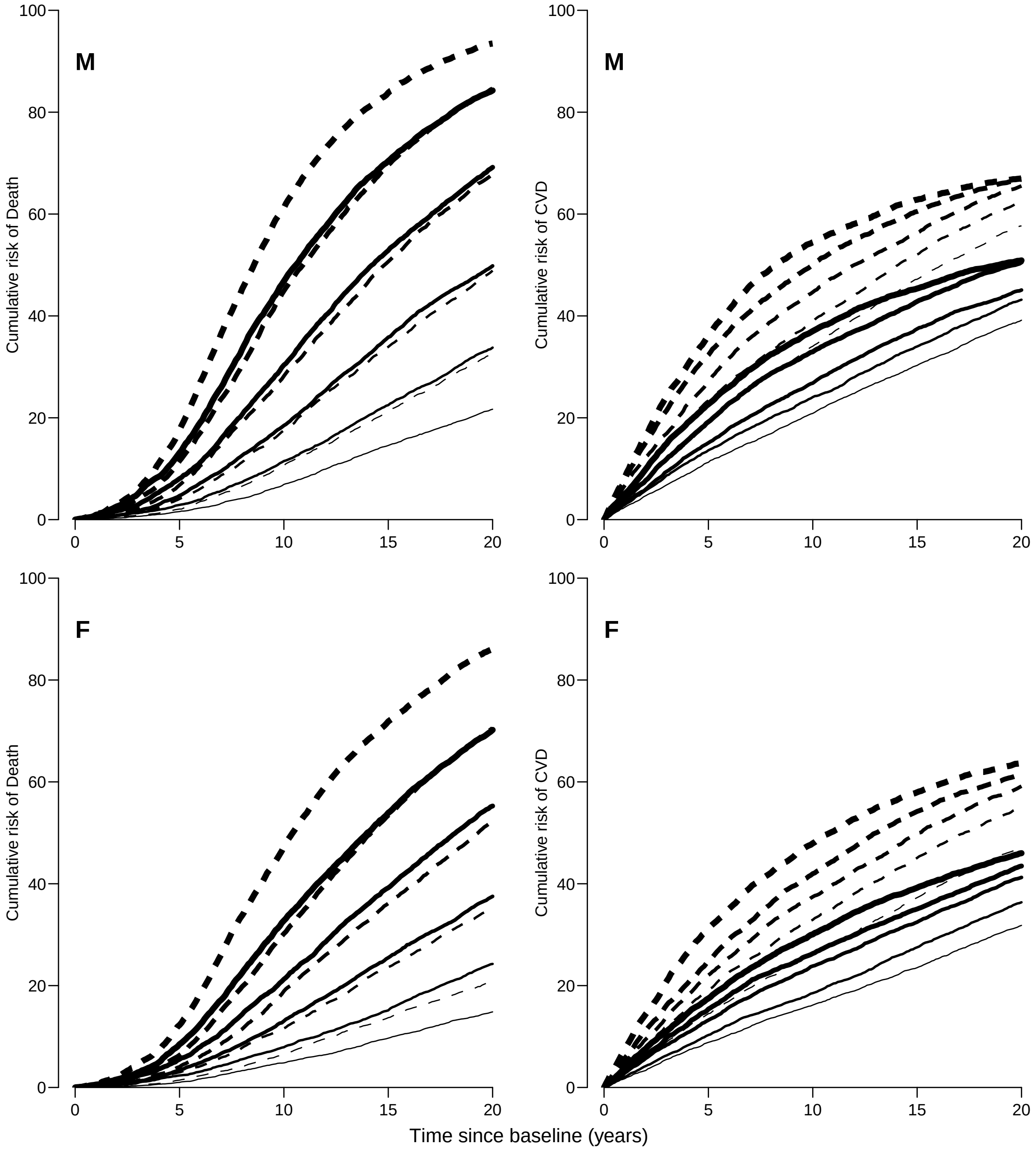

ESM Figure 7: *Cumulative risks of death and CVD by sex and age (45, 50, 55, 60 and 65) at entry. Curves for the 45 age-class are lowest. The full lines are for the intensive group, and the broken lines for the conventional group.*

7 Microvascular events at clinical visits

At each clinical visit the patients' status with respect to retinopathy, neuropathy and albuminuria were recorded. Since persons are only seen at visits, it is only known that a particular event occurred some time between two visits, not the exact time of occurrence. This is called *interval censoring*.

Interval censored event times were handled by simulating a random time for the event and then use these imputed data in a standard analysis of the hazard of the events. We assessed the contribution to the variance of estimates from this imputation by performing analyses of data sets from many different imputations. The contribution to uncertainty from the imputations was less than 1% of the s.e. of the estimates, so we could safely ignore the imputation as a source of variation in the results.

We fitted statistical models for hazard rates of (further) complication *and* mortality from each state of complication. With such models it is possible to compute the probability that a person is in a given state at given time, including the probability that a person were in a given state at time of death. These probabilities are derived for patient populations with age- and sex composition as the Steno 2 population, only differing in treatment allocation. Hence it is possible to judge the cumulative fraction of patients that will reach a given level of complication during the first 20 years of follow-up subject to either intensive or conventional treatment.

7.1 Retinopathy

At each visit patients were classified with respect to retinopathy as one of none / minimal / moderate / pre-proliferative / proliferative / photocoagulation. Deaths were recorded by the state of retinopathy the person was in at the time of death, defined as the state recorded at last visit before death.

Some patients improved their status between visits, so we estimated in two different scenarios, one where improvement was accepted, and one where only progression was allowed, the latter corresponding to scoring patients as in "the worst state seen so far".

We fitted separate models for the progression (and improvement) of retinopathy using time since baseline, age (at baseline) and sex as covariates and assuming proportional hazards between allocation groups (intensive/conventional) and between states of retinopathy. Similar models were fitted for mortality from each of the states.

Figure 8 shows the number of transitions between retinopathy states and death when improvement of retinopathy is allowed, and figure 9 the same with only progression is allowed. The associated HRs between intensive and conventional from the two approaches are shown in table 6.

ESM Table 6: *HRs of retinopathy improvement, progression and death between intensive and conventional groups. All HRs are controlled for attained retinopathy state.*

	Both directions			Only progression		
	HR	95% CI	P	HR	95% CI	P
Unadjusted						
Improvement	3.40	(1.41–8.23)	0.007			
Progression	0.73	(0.56–0.96)	0.024	0.67	(0.51–0.89)	0.005
Mortality	0.56	(0.36–0.87)	0.010	0.56	(0.37–0.86)	0.008
Adjusted for age						
Improvement	3.46	(1.44–8.31)	0.006			
Progression	0.74	(0.56–0.96)	0.026	0.67	(0.51–0.88)	0.004
Mortality	0.58	(0.38–0.90)	0.014	0.55	(0.36–0.85)	0.007

We used the estimated transition rates (the arrows shown in figures 8 and 9) to predict the probability of being in any one of the states given the initial state distribution; these are shown in figures 10 and 11.

The overall prevalence of at least moderate retinopathy at baseline was 15% overall. Using the model where improvement is allowed, we see that prevalence of at least moderate retinopathy over 20 years increase to 34% in the intensive-therapy group, but to 49% in the conventional. Using the progression-only model predicts increases to 46% respectively 53%.

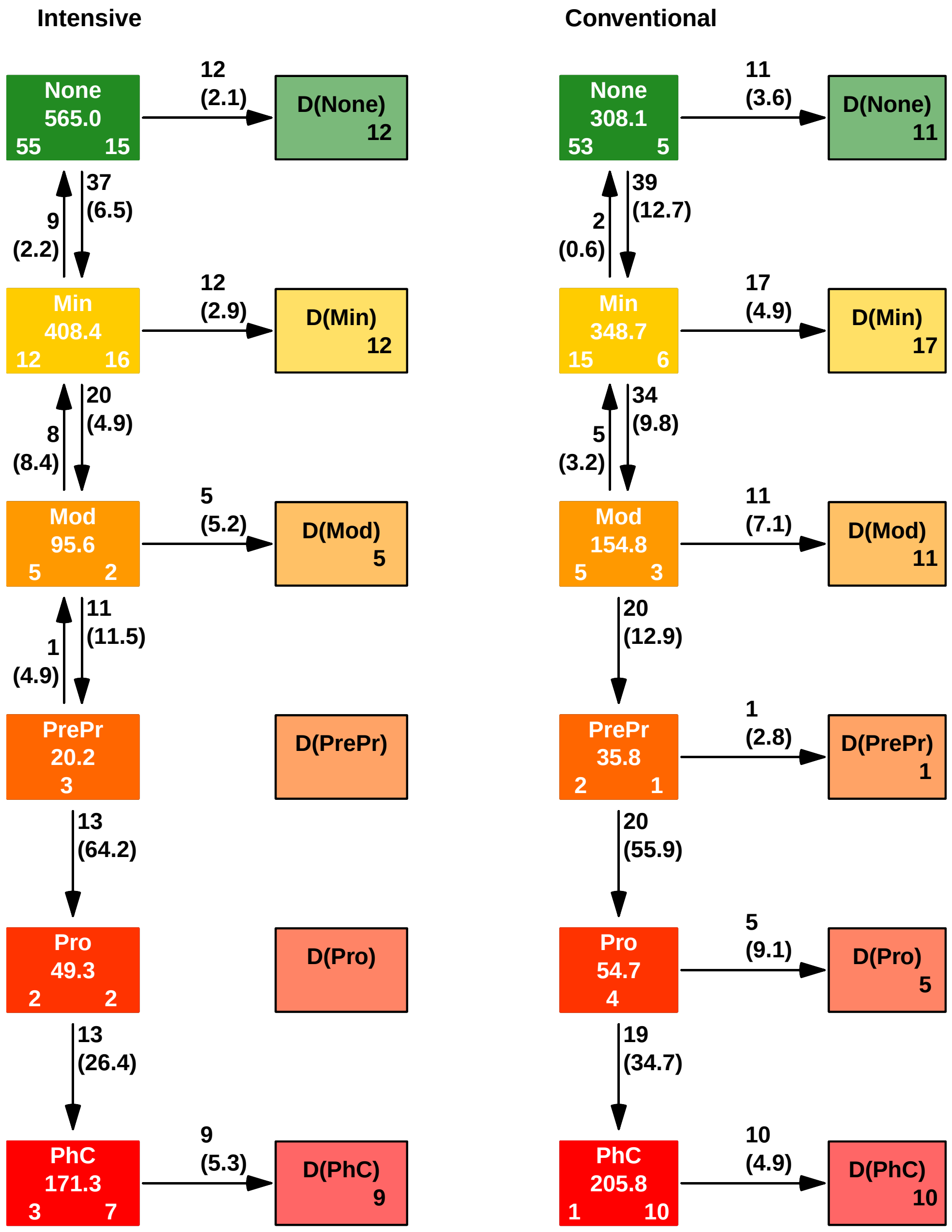

ESM Figure 8: *Transitions between states of retinopathy and death, allowing improvements in retinopathy status (except from the photocoagulation state). Only transitions between states actually occurring are shown. States are as described in section 3 on p. 6.*
The number in the center of the boxes is the person-years (PY), the numbers at the bottom is the number of patients starting, resp. ending their follow-up in each box (state), and the numbers on the arrows are the number of transitions and the overall transition rates per 100 PY.

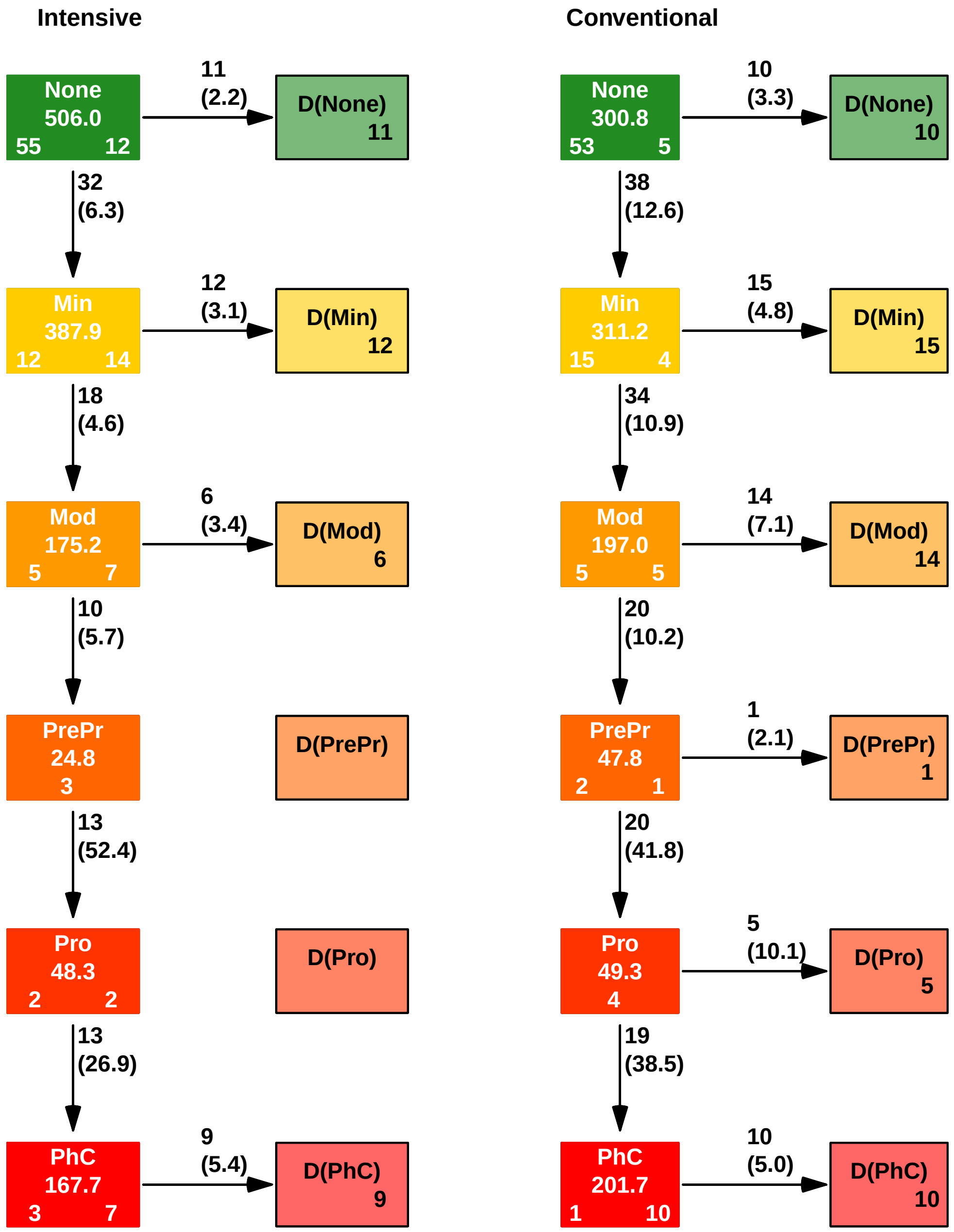

ESM Figure 9: *Transitions between states of retinopathy and death, allowing only deterioration in retinopathy status. The number in the center of the boxes is the person-years (PY), the numbers at the bottom is the number of patients starting, resp. ending their follow-up in each box (state), and the numbers on the arrows are the number of transitions and the overall transition rates per 100 PY.*

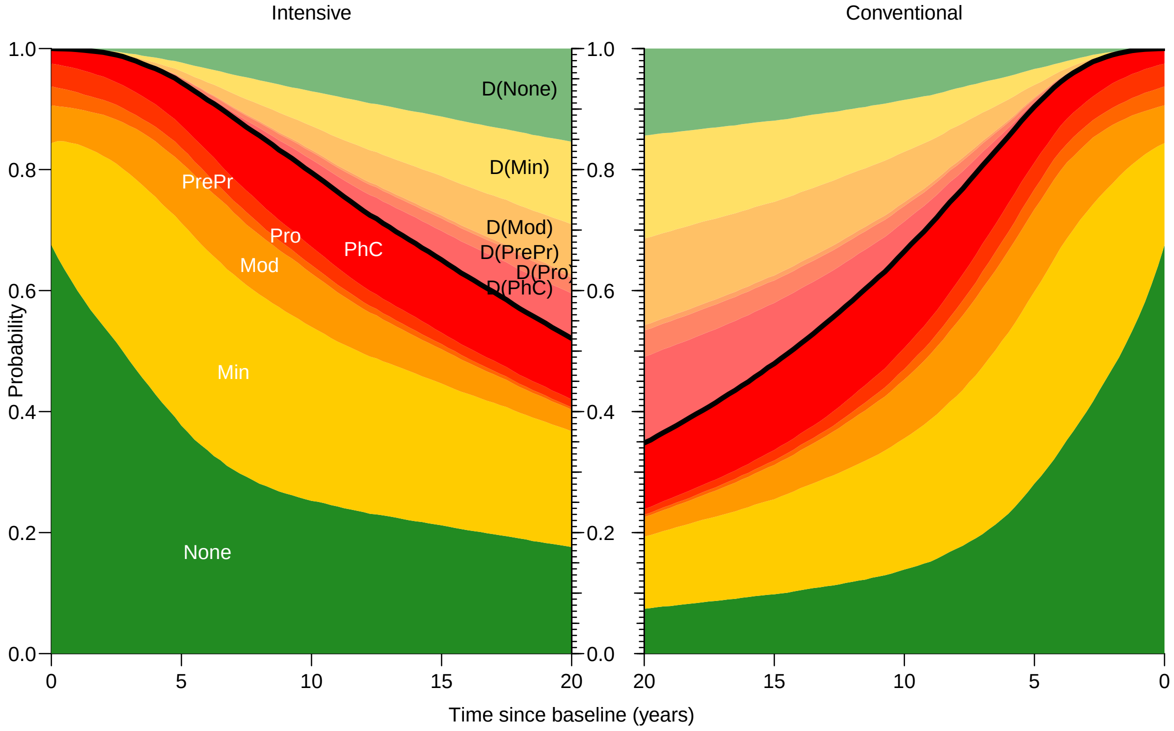

ESM Figure 10: *Distribution of states from a model where both improvement and deterioration of retinopathy are allowed: Patients with age- sex- and retinopathy score distribution as in Steno 2 study. The black line is the survival curve, modeling and coloring corresponds to the one in figure* 8.

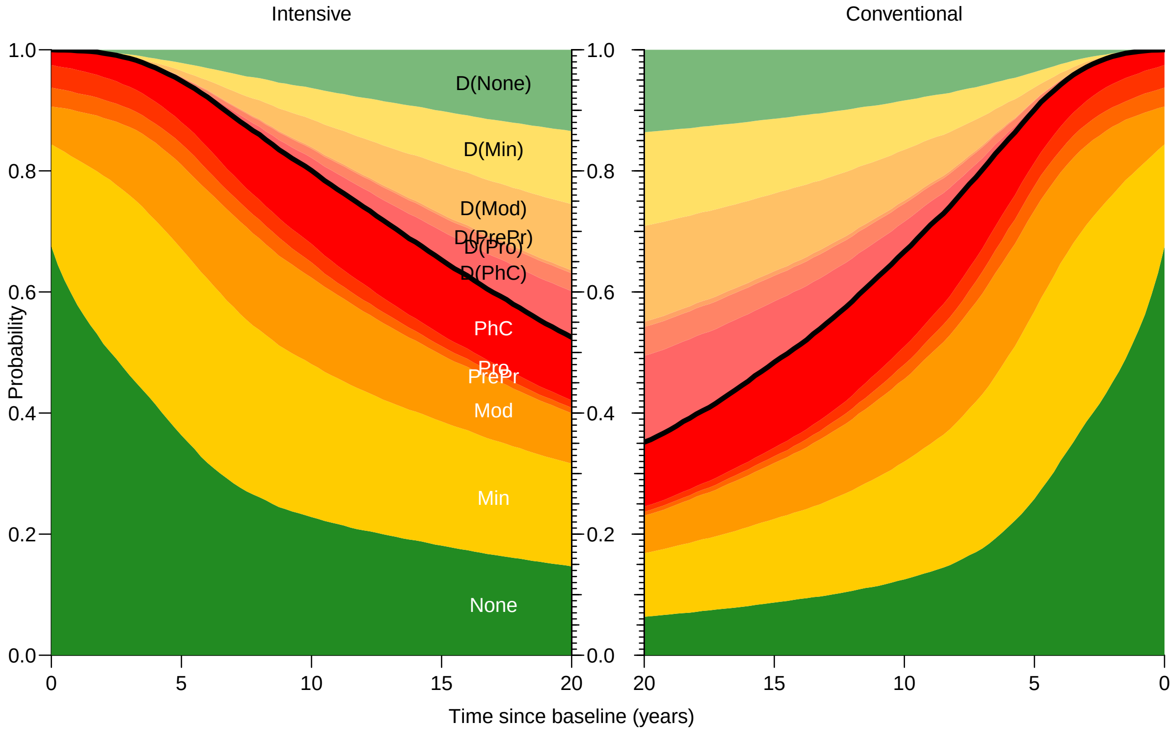

ESM Figure 11: *Distribution of retinopathy, from a model with only progression possible, otherwise as figure* 10.

7.2 Neuropathy

Patients were classified with respect to progression of autonomic or peripheral neuropathy since baseline. Progression of autonomic neuropathy was assessed from the 2nd visit, but peripheral only from the 3rd visit. Hence all patients are in the "None" state at baseline — "None" meaning no progression of neuropathy since baseline. Only patients with at least one assessment of autonomic ($n = 155$) or peripheral ($n = 152$) were included in the analyses.

For both types of neuropathy we imputed progression times and analyzed data by Poisson regression models with events as response and log-person-years as offset, controlling for age and sex. Together with models for the mortality from states with and without progression these (see figure 12) were used to predict the cumulative risk of neuropathy progression.

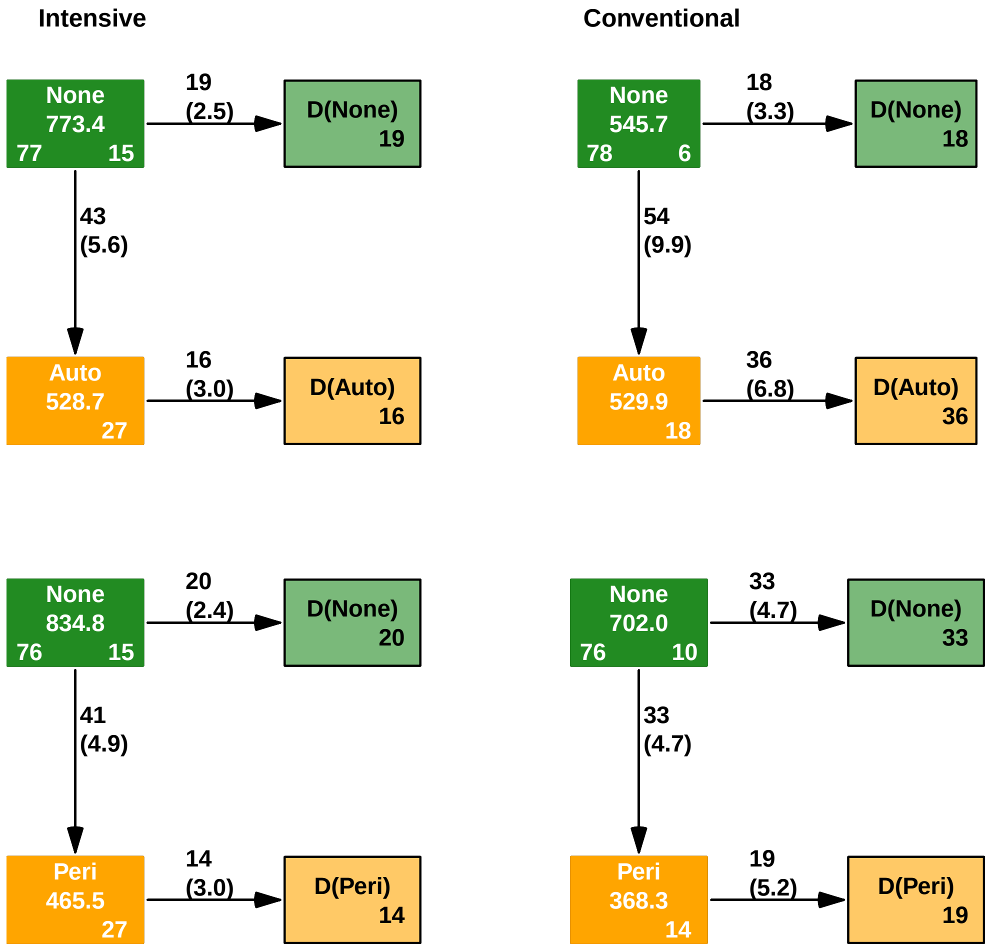

ESM Figure 12: *Transitions between states of neuropathy progression. The number in the center of the boxes is the person-years (PY), the numbers at the bottom is the number of patients starting, resp. ending their follow-up in each box (state), and the numbers on the arrows are the number of transitions and the overall transition rates per 100 PY. Note that the total number of patients is not 160; only patients with at least one assessment were included. Upper panels: autonomic neuropathy, lower panels: peripheral neuropathy.*

7.2.1 Autonomic neuropathy

The intensive group had a smaller rate of autonomic neuropathy progression, (HR= 0.59, (0.40;0.89), p=0.011), there was no indication of non-proportional hazards, and mortality was not different between persons with and without autonomic neuropathy (HR 0.97 (0.62;1.52) p=0.890). We used the proportional hazards model for mortality to predict the fraction of persons with progression of autonomic neuropathy, as seen in figure 13

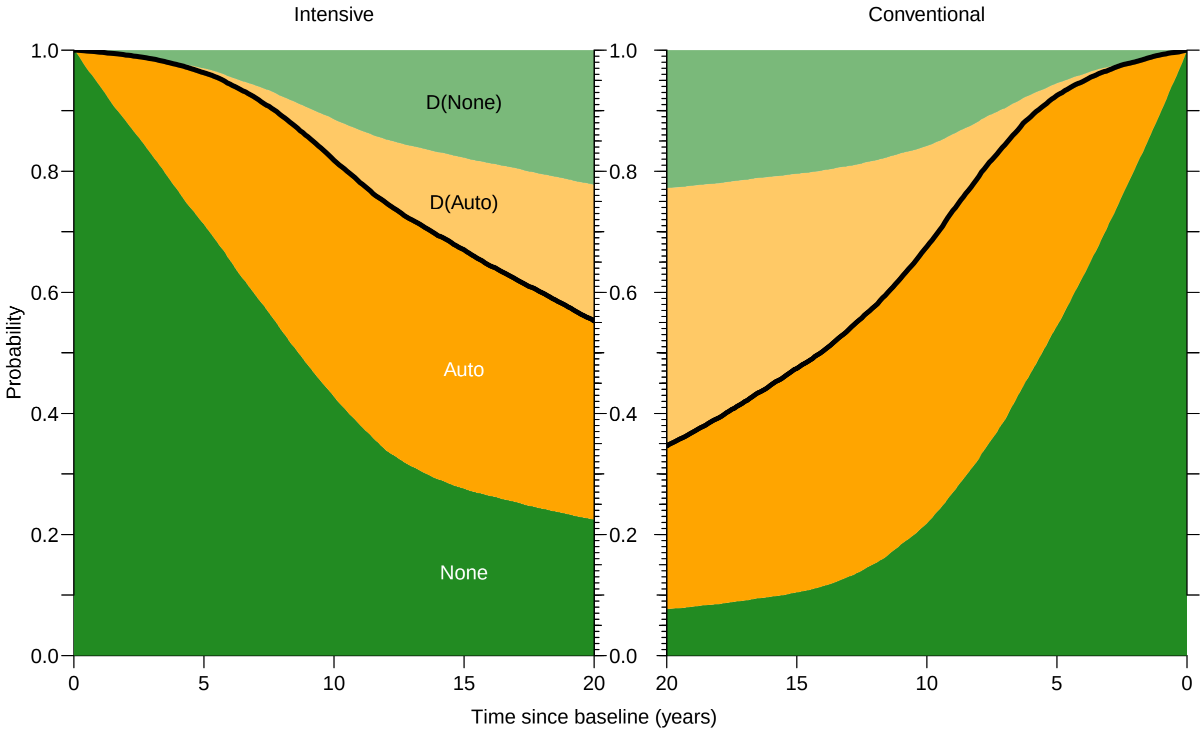

ESM Figure 13: *Fraction of patients with progression of autonomic neuropathy, based on models for progression of autonomic neuropathy and for mortality.*

There are 59% in the intensive group and 68% in the conventional group that have progression of autonomic neuropathy (13).

7.2.2 Peripheral neuropathy

There was no difference between the intervention groups in the occurrence rate of peripheral neuropathy (HR 1.12 (0.71;1.77), p=0.630), and no difference in mortality between persons with and without peripheral neuropathy (HR 0.91 (0.58-1.44), p=0.704).

We used the models with proportional hazards to predict the fraction of persons with progression of peripheral neuropathy, as seen in figure 14. There is a higher fraction of persons that during the first 20 years progress in peripheral neuropathy in the intensive group than in the conventional group (53% vs. 45%); this is attributable to the smaller mortality in the intensive group.

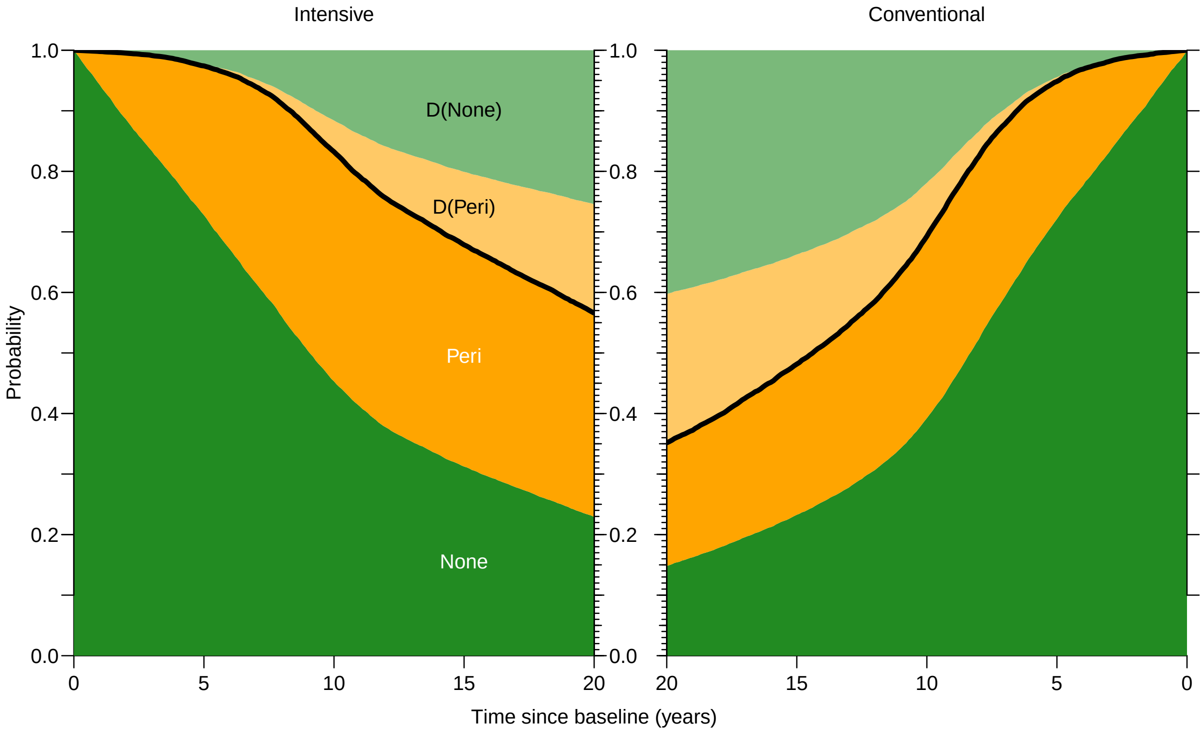

ESM Figure 14: *Fraction of patients with progression of peripheral neuropathy (orange), based on models for progression of peripheral neuropathy and for mortality.*

7.3 Albuminuria

It was an inclusion criterion to be micro-albuminuric, so all patients are in this state at the first visit; at later visits they were classified as normo / micro / macro-albuminuric. We used a model where persons could move between the three states; figure 15 shows the possible states and the number of transitions for the two randomization groups separately.

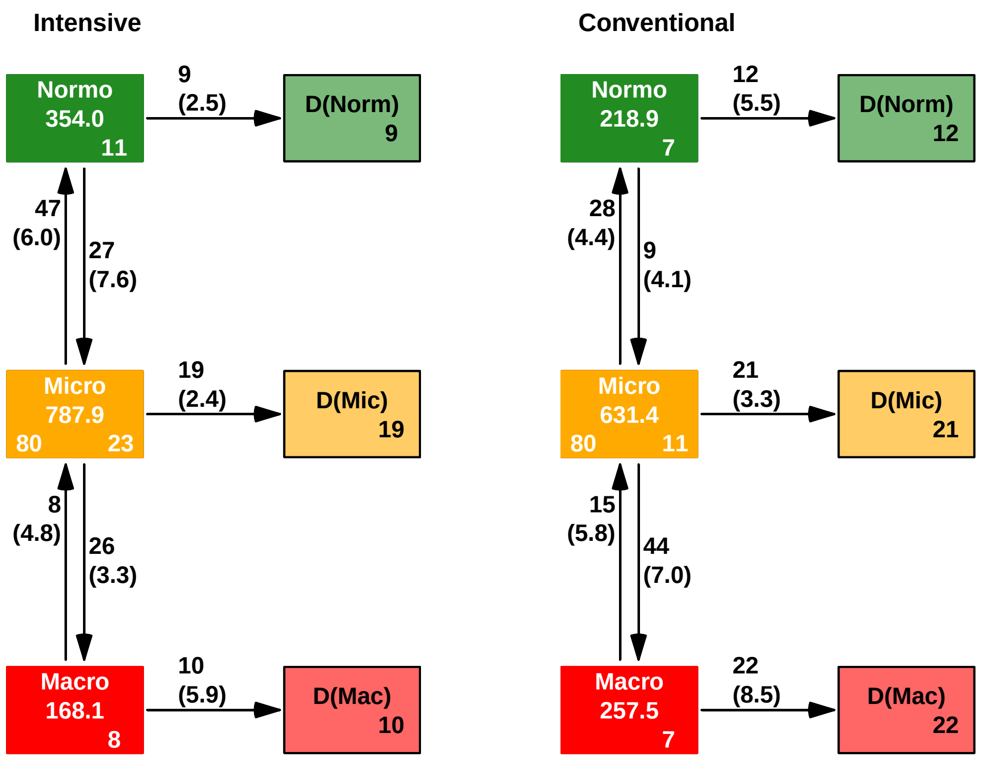

ESM Figure 15: *Transitions between albuminuria states. The number in the center of the boxes is the person-years (PY), the numbers at the bottom is the number of patients starting, resp. ending their follow-up in each box (state), and the numbers on the arrows are the number of transitions and the overall transition rates per 100 PY.*

We fitted models for transition rates for increasing resp. decreasing albuminuric state, with different intervention effects and a model for mortality with the same effect of intervention on all rates; the estimates are shown in figure 16.

From figure 16 we see that compared to the conventional group, the intensive group have higher (though not significantly so) rates of transition to better status, and significantly lower transition rates from micro-albuminuria to macro-albuminuria

We used these models to predict the fraction of the patients that would be in different states at different times, as seen in figure 17

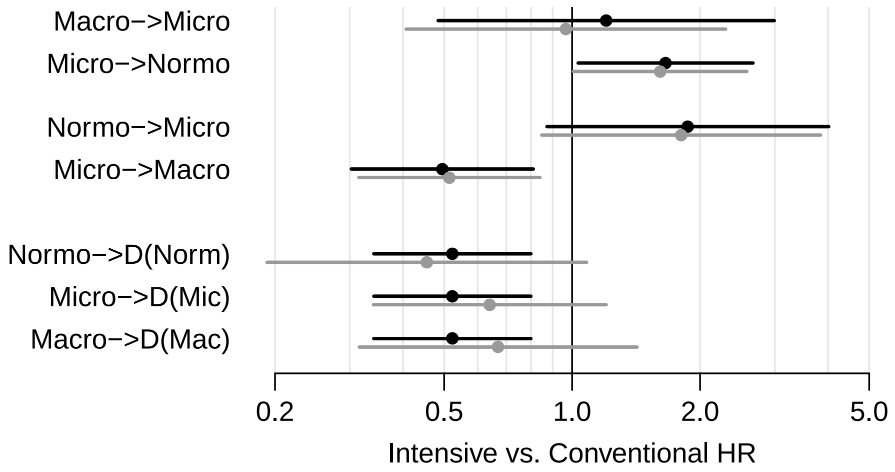

ESM Figure 16: *Hazard ratios between intensive and conventional for the different transitions between albuminuria states and death — there is one HR for each of the arrows in figure 15. The gray lines are HR estimates from a Cox-model allowing different allocation effects on mortality from different states.*

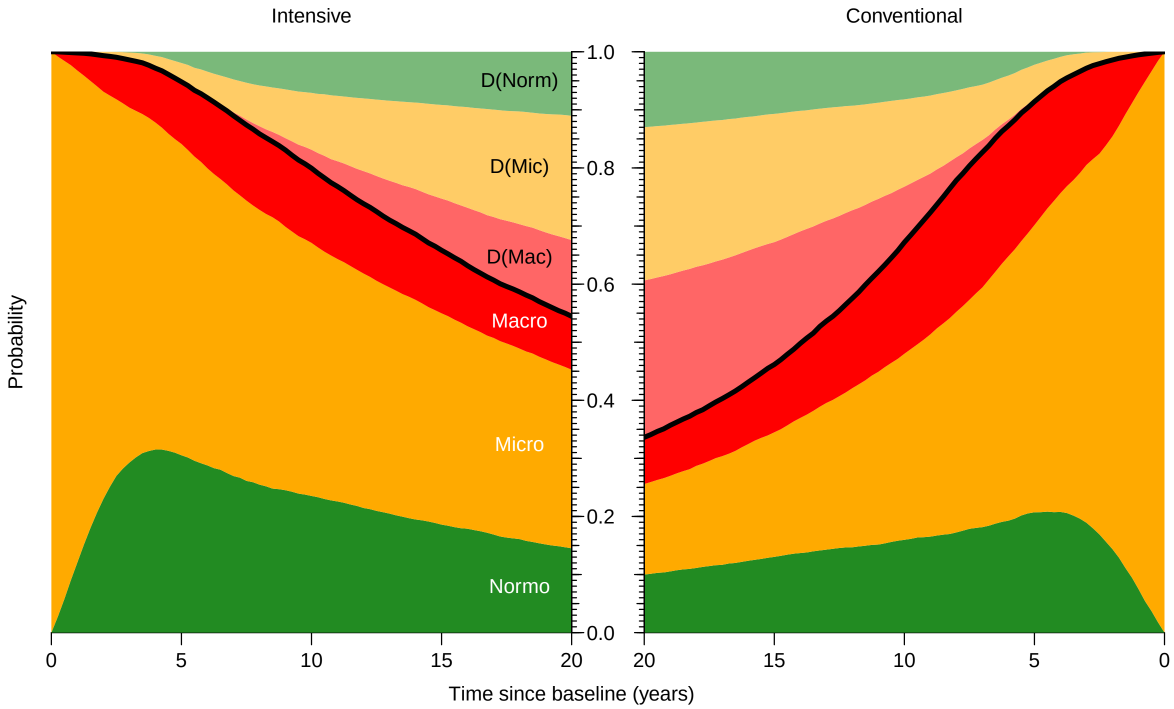

ESM Figure 17: *Fraction of patients in different states of albuminuria by time since baseline. The black curves are the survival curves and the colored areas show the fraction of the patients in each state by time. Note that all persons start in the "Micro" state by study design.*

References

[1] R Core Team. *R: A Language and Environment for Statistical Computing*. R Foundation for Statistical Computing, Vienna, Austria, 2016.

[2] Martyn Plummer and Bendix Carstensen. Lexis: An R class for epidemiological studies with long-term follow-up. *Journal of Statistical Software*, 38(5):1–12, 1 2011.

[3] Bendix Carstensen and Martyn Plummer. Using Lexis objects for multi-state models in R. *Journal of Statistical Software*, 38(6):1–18, 1 2011.

[4] Bendix Carstensen, Martyn Plummer, Esa Laara, and Michael Hills et. al. *Epi: A package for statistical analysis in epidemiology.*, 2016. R package version 2.5.
